# Artificial intelligence methods to detect heart failure with preserved ejection fraction within electronic health records: an equitable disease detection model

**DOI:** 10.1093/ehjdh/ztaf107

**Published:** 2025-09-16

**Authors:** Jack Wu, Dhruva Biswas, Samuel Brown, Matthew Ryan, Brett S Bernstein, Brian Tam To, Tom Searle, Maleeha Rizvi, Natalie Fairhurst, George Kaye, Ranu Baral, Dhanushan Vijayakumar, Daksh Mehta, Narbeh Melikian, Daniel Sado, Gerald Carr-White, Phil Chowienczyk, James Teo, Richard J B Dobson, Daniel I Bromage, Thomas F Lüscher, Ali Vazir, Theresa A McDonagh, Jessica Webb, Ajay M Shah, Kevin O’Gallagher

**Affiliations:** School of Cardiovascular and Metabolic Medicine & Sciences, British Heart Foundation Centre of Research Excellence, King’s College London, London SE5 9NU, UK; School of Cardiovascular and Metabolic Medicine & Sciences, British Heart Foundation Centre of Research Excellence, King’s College London, London SE5 9NU, UK; Section of Cardiovascular Medicine, Department of Internal Medicine, Yale School of Medicine, New Haven, CT 06510, USA; School of Cardiovascular and Metabolic Medicine & Sciences, British Heart Foundation Centre of Research Excellence, King’s College London, London SE5 9NU, UK; Cardiovascular Department, King’s College Hospital NHS Foundation Trust, London SE5 9RS, UK; School of Cardiovascular and Metabolic Medicine & Sciences, British Heart Foundation Centre of Research Excellence, King’s College London, London SE5 9NU, UK; Guy’s and St Thomas’ Hospital, Guy’s and St Thomas’ NHS Foundation Trust, London SE1 7EH, UK; School of Cardiovascular and Metabolic Medicine & Sciences, British Heart Foundation Centre of Research Excellence, King’s College London, London SE5 9NU, UK; Cardiovascular Department, King’s College Hospital NHS Foundation Trust, London SE5 9RS, UK; Cardiovascular Department, King’s College Hospital NHS Foundation Trust, London SE5 9RS, UK; Department of Biostatistics and Health Informatics, Institute of Psychiatry, Psychology and Neuroscience, King’s College London, London SE5 8AB, UK; School of Cardiovascular and Metabolic Medicine & Sciences, British Heart Foundation Centre of Research Excellence, King’s College London, London SE5 9NU, UK; Guy’s and St Thomas’ Hospital, Guy’s and St Thomas’ NHS Foundation Trust, London SE1 7EH, UK; Cardiovascular Department, King’s College Hospital NHS Foundation Trust, London SE5 9RS, UK; Cardiovascular Department, King’s College Hospital NHS Foundation Trust, London SE5 9RS, UK; Cardiovascular Department, King’s College Hospital NHS Foundation Trust, London SE5 9RS, UK; King’s College London GKT School of Medical Education, London WC2R 2LS, UK; King’s College London GKT School of Medical Education, London WC2R 2LS, UK; School of Cardiovascular and Metabolic Medicine & Sciences, British Heart Foundation Centre of Research Excellence, King’s College London, London SE5 9NU, UK; Cardiovascular Department, King’s College Hospital NHS Foundation Trust, London SE5 9RS, UK; School of Cardiovascular and Metabolic Medicine & Sciences, British Heart Foundation Centre of Research Excellence, King’s College London, London SE5 9NU, UK; Cardiovascular Department, King’s College Hospital NHS Foundation Trust, London SE5 9RS, UK; Guy’s and St Thomas’ Hospital, Guy’s and St Thomas’ NHS Foundation Trust, London SE1 7EH, UK; Guy’s and St Thomas’ Hospital, Guy’s and St Thomas’ NHS Foundation Trust, London SE1 7EH, UK; Cardiovascular Department, King’s College Hospital NHS Foundation Trust, London SE5 9RS, UK; Department of Biostatistics and Health Informatics, Institute of Psychiatry, Psychology and Neuroscience, King’s College London, London SE5 8AB, UK; Department of Biostatistics and Health Informatics, Institute of Psychiatry, Psychology and Neuroscience, King’s College London, London SE5 8AB, UK; School of Cardiovascular and Metabolic Medicine & Sciences, British Heart Foundation Centre of Research Excellence, King’s College London, London SE5 9NU, UK; Cardiovascular Department, King’s College Hospital NHS Foundation Trust, London SE5 9RS, UK; Guy’s and St Thomas’ Hospital, Guy’s and St Thomas’ NHS Foundation Trust, London SE1 7EH, UK; Guy’s and St Thomas’ Hospital, Guy’s and St Thomas’ NHS Foundation Trust, London SE1 7EH, UK; School of Cardiovascular and Metabolic Medicine & Sciences, British Heart Foundation Centre of Research Excellence, King’s College London, London SE5 9NU, UK; Cardiovascular Department, King’s College Hospital NHS Foundation Trust, London SE5 9RS, UK; Guy’s and St Thomas’ Hospital, Guy’s and St Thomas’ NHS Foundation Trust, London SE1 7EH, UK; School of Cardiovascular and Metabolic Medicine & Sciences, British Heart Foundation Centre of Research Excellence, King’s College London, London SE5 9NU, UK; Cardiovascular Department, King’s College Hospital NHS Foundation Trust, London SE5 9RS, UK; School of Cardiovascular and Metabolic Medicine & Sciences, British Heart Foundation Centre of Research Excellence, King’s College London, London SE5 9NU, UK; Cardiovascular Department, King’s College Hospital NHS Foundation Trust, London SE5 9RS, UK

**Keywords:** Heart failure with preserved ejection fraction, Prediction model, Electronic health records

## Abstract

**Aims:**

Heart failure with preserved ejection fraction (HFpEF) accounts for approximately half of all heart failure cases, with high levels of morbidity and mortality. However, many patients who meet diagnostic criteria for HFpEF do not have a documented diagnosis, particularly in non-White populations where conventional risk scores may underestimate risk. Our aim was to develop and validate a diagnostic prediction model to detect HFpEF based on ESC criteria, AIM-HFpEF.

**Methods and results:**

We applied natural language processing (NLP) and machine learning methods to routinely collected electronic health record (EHR) data from a tertiary centre hospital trust in London, UK, to derive the AIM-HFpEF model. We then externally validated the model and performed benchmarking against existing HFpEF prediction models (H2FPEF and HFpEF-ABA) for diagnostic power on the entire external cohort and in patients of non-White ethnicity and patients from areas of increased socioeconomic deprivation. An XGBoost model combining demographic, clinical, and echocardiogram data showed strong diagnostic performance in the derivation dataset [*n* = 3173, AUC = 0.88, (95% CI, 0.85–0.91)] and validation cohort [*n* = 5383, AUC: 0.88 (95% CI, 0.86–0.90)]. Diagnostic performance was maintained in patients of non-White ethnicity [AUC = 0.89 (95% CI, 0.85–0.93)] and patients from areas of high socioeconomic deprivation [AUC = 0.90 (95% CI, 0.85–0.95)]. In contrast, AIM-HFpEF demonstrated favourable performance relative to the H2FPEF and HFpEF-ABA models. AIM-HFpEF model probabilities were associated with an increased risk of death, hospitalization, and stroke in the external validation cohort (*P* < 0.001, *P* = 0.01, *P* < 0.001, respectively, for highest vs. middle tertile).

**Conclusion:**

AIM-HFpEF represents a validated equitable diagnostic model for HFpEF, which can be embedded within an EHR to allow for fully automated HFpEF detection.

## Graphical Abstract

**Graphical Abstract:**
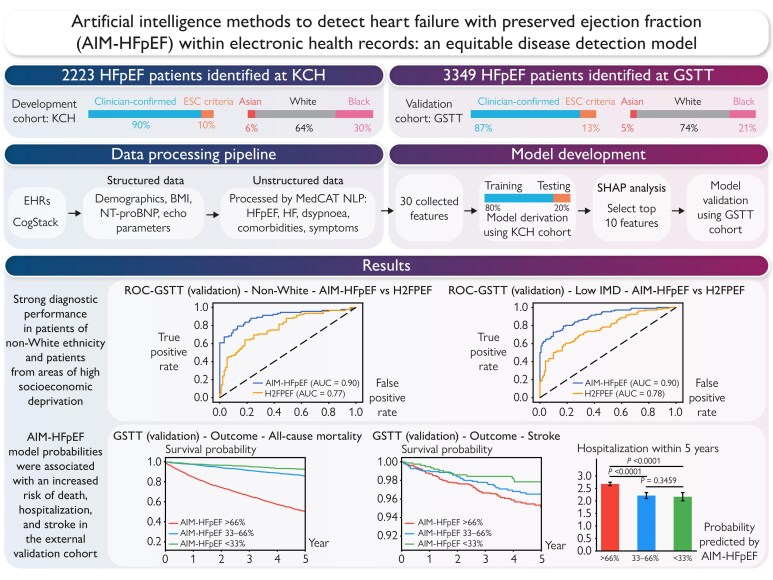

## Introduction

Heart failure with preserved ejection fraction (HFpEF) accounts for approximately half of all heart failure (HF) cases and is associated with significant healthcare costs, morbidity, and mortality. Early diagnosis is important and has prognostic benefit.^1^ However, despite the high prevalence of HFpEF, most cases are not formally recorded as such in the clinical record.^2^ A recent study of community-based adults suggests that HFpEF remains substantially under-recognized, particularly among individuals with obesity and unexplained dyspnoea.^3^ Therefore a high proportion of patients do not benefit from specialist cardiology care, evidence-based therapies, and eventually improved outcomes.^4,5^

Strategies have been developed to improve HFpEF detection, including the H2FPEF scoring system.^6^ The H2FPEF score is validated for the diagnosis of HFpEF, but requires an a priori suspicion of HFpEF. More recently, the HFpEF-ABA score, based on clinical features alone with no cardiac imaging features, has been developed as a screening tool to identify possible HFpEF cases and guide the need for specialist cardiac imaging and clinical evaluation.^7^

HFpEF is disproportionately under-diagnosed particularly in patients of non-White race and ethnicity.^8^ Indeed, Black and Asian patients with HFpEF have different patterns of comorbidity to White patients, including features used in current HFpEF diagnostic systems such as atrial fibrillation (AF) and body mass index.

The widespread deployment of electronic health record (EHR) platforms provides the potential to enable access to a wide range of routinely collected clinical data in a fraction of the time taken to perform manual case record completion. EHR-based diagnostic approaches lend themselves to automation, removing the need for clinician-initiated suspicion of disease and therefore potentially decreasing the risk of bias. Moreover, advances in artificial intelligence (AI) methods allow for the capture of both structured and unstructured data, including AI-based detection of clinical concepts from free text via natural language processing (NLP)^9–12^ with potentially less data missingness in populations less engaged with health services. We have previously used these methods to detect HFpEF from the EHR, finding that <10% of all cases of HFpEF have a clinician-assigned diagnosis, while the remaining 90% had no formal documentation of the condition in their records, despite meeting diagnostic criteria.^2^

The aim of this study was to develop and externally validate a scalable and automated prediction model for detecting HFpEF by applying AI methods to routinely collected data from the EHR of two independent, ethnically diverse cohorts of HFpEF patients, including a representative distribution of cases of those with a clinician-assigned diagnosis (HFpEF – Confirmed) and those meeting ESC diagnostic criteria without a documented diagnosis (HFpEF – ESC Criteria). A further aim was to assess the performance of the prediction model across racial and ethnicity groups and in patients from socially deprived areas. The proposed model can serve as an efficient and routinely executable support tool in the EHR system to flag patients with a high probability of HFpEF for further evaluation, which may include advanced diagnostic techniques like invasive haemodynamic (exercise) testing (RHC). Our approach prioritizes scalability and automation to identify at-risk patients who might otherwise remain undetected.

## Methods

### Ethical considerations

This project operated under London South-East Research Ethics Committee approval (18/LO/2048) granted to the King’s Electronic Records Research Interface (KERRI) and London Dulwich Research Ethics Committee approval (19/LO/1957), which did not require written informed patient consent. The study complies with the Declaration of Helsinki. Patients were consulted on the study via a dedicated patient and public involvement (PPI) meeting held during the study design phase. A formal protocol was not published and the study was not registered.

### Participating centres

King’s College Hospital NHS Foundation Trust (KCH) and Guy’s and St Thomas’ NHS Foundation Trust (GSTT) are two large, multi-site tertiary hospitals in London, UK, providing specialist cardiology services and a dedicated HF service open to referral by any physician.

### Study design and inclusion criteria

**Figure 1:**
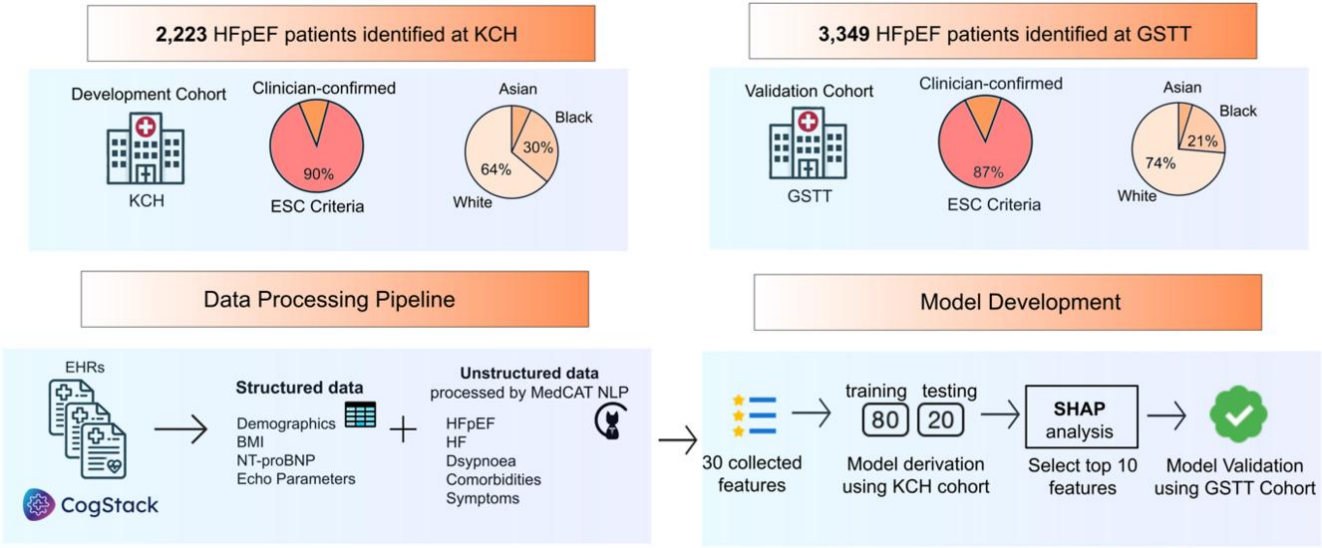
Schematic diagram of the study.

At each participating centre, we established a registry comprising a retrospective anonymized database of adult patients with a clinical diagnosis of HF documented within the EHR between 2010 and 2022. *Figure 1* shows the schematic diagram of the study. Patients were included if they had two or more mentions of a ‘heart failure’ diagnosis in the clinical text as determined by a well-validated NLP pipeline^13–16^ described in Supplementary Document  *S1*. Specifically, a random sample of 100 HFpEF patients identified by the NLP pipeline were manually validated for this study and 100% of them were true positive. Patients were included regardless of nature of clinical episode (inpatient or outpatient). Both structured and unstructured portions of the echocardiogram report were used to extract left ventricular ejection fraction (LVEF) data of patients and other relevant echocardiographic parameters. Patients with a clinical diagnosis of HF and LVEF ≥50% were categorized into one of the following two groups:

1. HFpEF – Confirmed: Clinician-assigned diagnosis of HFpEF
2. HFpEF – ESC Criteria: Patients with HF, LVEF ≥50%, and imaging/biochemical evidence of diastolic dysfunction meeting the ESC diagnostic criteria^17^ (objective evidence of cardiac structural and functional abnormalities including elevated E/e’, increased LA volume index, raised NTproBNP, or pulmonary hypertension) but who have not received a HFpEF diagnosis.

Patients were excluded if at any point they had an echocardiogram with LVEF <50%. Patients with a clinical diagnosis of HF and LVEF ≥50% on echocardiography, but not meeting ESC diagnostic criteria and who have not received a HFpEF diagnosis, were excluded, as were patients with an alternative diagnosis (severe valvular heart disease, hypertrophic cardiomyopathy, restrictive cardiomyopathy, constrictive pericarditis, and cardiac amyloidosis). Supplementary material online, *Table S1* contains the SNOMED terms used in the classification of included and excluded patients. Patients needed to have the echocardiogram report within 1 year of clinical diagnosis of HF; otherwise, they were excluded. Note that the HFpEF – ESC Criteria patients, although having HF diagnosis (with positive mentions of HF in their EHRs), were never given a formal HFpEF diagnosis. This gap can have important implications for care and outcomes, as without a recognized diagnosis, and potentially consequent coding in the EHR, guideline-directed therapies aimed at improving outcomes for HFpEF may not be considered, both in hospital and primary care settings.

At each site we also established a registry of non-cardiac dyspnoea patients to act as control groups. Patients in the control group needed (1) two or more mentions of dyspnoea in the clinical text as determined by NLP; (2) an echocardiogram report (LVEF ≥ 50%) within 1 year of the dyspnoea mentions; and (3) no mentions of HF or HFpEF diagnosis in the clinical text. Patients with severe valvular heart disease, hypertrophic cardiomyopathy, restrictive cardiomyopathy, constrictive pericarditis, or cardiac amyloidosis were also excluded from the control group.

Although invasive haemodynamic (exercise) testing (RHC) remains a reference method for diagnosing HFpEF, it is rarely performed in large, routine care cohorts. By using the ESC criteria to identify the HFpEF – ESC Criteria group (i.e. HF patients without documented HFpEF diagnosis), our method therefore provides a pragmatic approach of detecting both clinically recognized and unrecognized HFpEF at scale within EHRs.

### Prediction model development

The dataset collected from KCH was used as the derivation cohort, with 80% of the data used for training and 20% for testing, while the GSTT dataset was used for external validation. All model development and evaluation were performed using Python version 3.10.12.

We derived prediction models using four different methods: logistic regression (LR), support vector machine (SVM), random forest (RF), and XGBoost (XGB). The machine learning models were built and evaluated using the ‘scikit-learn’ (for LR, SVM and RF) and ‘xgboost’ (for XGB) python packages. Hyperparameter tuning for each model was performed through grid search and 10-fold cross-validation using the training dataset only. The ‘StandardScaler’ from ‘scikit-learn.preprocessing’ was applied to the data before model training for consistent scaling across echocardiograph parameter features for LR, SVM, and RF models. Mean imputation was applied to the missing values for the three (LR, SVM, and RF) machine learning models, missForest imputation was also tested, and the correlation of the predicted probabilities from both methods were >98%. As XGB can handle scaling and missing values internally, we did not perform scaling and imputation for the XGB model. Model evaluation was based on metrics including accuracy, precision, recall, F1 score, and AUC-ROC, calculated using functions from ‘scikit-learn.metrics’. The comparison of AUCs was performed using a python implementation of the DeLong test.^18^ We selected the model, which had the best overall performance for the Results section while the performances for non-selected models are reported in Supplementary material online, *Table S2*.

### Feature engineering

We aimed to produce a model based on the parameters included in the ESC criteria for diagnosing HFpEF. Features representing key demographic, echocardiographic, comorbid, and symptomatic factors commonly associated with HFpEF are used to construct the model, and they are extracted from both structured and unstructured data. Features from structured data include age, sex, BMI, NTproBNP, and echocardiographic parameters [E/e’, LA volume, LA volume indexed, IVSd, LVPWD, LVEDD, LV V1 max, LV V1 max PG, LV mass, LV mass indexed, and pulmonary artery systolic pressure (PASP)], while comorbidities (diabetes mellitus type 1, diabetes mellitus type 2, ischaemic heart disease, myocardial infarction, cerebrovascular accident, hypertensive disorder, transient ischaemic attack, AF, pulmonary hypertension, kidney disease, and angina) and symptoms (dyspnoea at rest, chest pain, dizziness, and syncope) were extracted from unstructured clinical notes through NLP using MedCAT^14^ within the CogStack platform.^19^ Echocardiographic parameters included in the ESC criteria were prioritized in the model, while other parameters with more than 30% missingness were excluded. Comorbidities and symptoms are represented as binary features, with a positive value indicating the presence of the comorbidity or symptom before the first mention of HF (for the HFpEF group) or dyspnoea (for the control group) in the EHR while a negative value indicates the absence of the condition. Dyspnoea was excluded as a feature in the model since it was part of the inclusion criteria for control patients, resulting in 100% of the control group presenting with dyspnoea. The full model includes 30 features in total and can be executed automatically on the CogStack platform using routinely collected EHR data.

A (Shapley Additive Explanations) SHAP analysis on the KCH training dataset was used to simplify the full prediction model. The top features, ranked by their SHAP values, were selected to construct a simplified prediction model. In the simplified model, diabetes mellitus type 1 and type 2 were combined into a single feature. The simplified model can be useful for manually inputting feature values in the form of an online application.

### Model explainability

The SHAP graph was plotted for the full model using the ‘shap’ python library (version 0.45.1)^20^ to show the importance and values of each feature contributing to the prediction outcome.

### Comparison with H2FPEF and HFpEF-ABA

For comparison with the H2FPEF score, we followed the more precise version of the H2FPEF probability using the formula provided in Reddy *et al*. (2018),^6^ specifically the online calculator from the Supplementary material online. The formula requires five key variables: BMI, AF, PASP, age, and filling pressure (E/e’). We also compared the results with the point-based version of the H2FPEF score (which requires six variables with antihypertensive drugs added) in Supplementary material online, *Table S2*. Patients with any missing values for these key variables were excluded from the comparison to ensure accurate probability estimation.

For comparison with the HFpEF-ABA score, we used the formula provided in Reddy *et al*. (2024),^7^ specifically from ‘Extended Data Table 4 Regression equations for clinical variable models’. This model requires three variables: age, BMI, and AF. Similar to the comparison with H2FPEF score, patients with missing values for any of these three variables were excluded from the comparison.

### Subgroup analysis

In the subgroup analysis, we examined two patient subgroups: (1) non-White individuals based on self-ascribed ethnicity^2^ and (2) those with low socioeconomic status, as assessed by the English Indices of Multiple Deprivation 2019 (IMD). The IMD was determined using postcodes of the patients. Patients were classified as having low IMD if their postcodes fell within the most deprived quintiles according to the national index. The model’s performance was evaluated separately in the two subgroups to assess potential variations in predictive accuracy for HFpEF based on ethnicity and socioeconomic status.

To better understand which patients are identified by the model but missed by current clinical practice, we performed a comparative analysis of key H2FPEF diagnostic features between the HFpEF – Confirmed and HFpEF – ESC Criteria groups.

### Prediction model output and calibration

The machine learning models output the probability of HFpEF for each patient. For consistency, the cut-off value for a positive HFpEF prediction is set at 0.5 when computing the accuracy, precision, and recall values in Supplementary material online, *Table S2*. The same threshold is used when comparing those metrics with the H2FPEF and HFpEF-ABA scores. In practice, the threshold can be adjusted according to individual treatment goals and preferences.^7^ The threshold does not affect the main comparison metric (i.e. ROC-AUC) in the study. Calibration of the prediction probabilities were assessed graphically using calibration curves produced by the ‘calibration_curve’ function in ‘scikit-learn’ package.

### Outcome data

Mortality data were obtained from death notification letters in the EHR system and the master demographic patient indices of the hospitals (synchronized with NHS Spine for demographics). Hospitalizations were estimated by the number of discharge notifications in the EHR in the study timeframe, i.e. 2010–2022. Diagnoses of myocardial infarction and stroke were recorded as outcome data if they occurred after the first mention of HF (for the HFpEF group) or dyspnoea (for the control group) in the EHR.

## Results

We identified 2223 HFpEF patients [231 (10%) Confirmed and 1992 (90%) ESC Criteria] at KCH and 3349 HFpEF patients [430 (13%) Confirmed and 2919 (87%) ESC Criteria] at GSTT. There were 950 and 2034 non-HF patients with dyspnoea at KCH and GSTT, respectively, for the control groups. The observed imbalance in the number of cases and controls reflects the real-world distribution of HFpEF cases and ‘non-cardiac dyspnoea’ controls within our dataset. This distribution was preserved intentionally to ensure the model remains representative of clinical practice, where HFpEF is often under-diagnosed. *Table 1* shows the baseline characteristics of the patients. Supplementary material online, *Figure S1* shows the number of enrolled and excluded patients with corresponding reasons.

**Table 1:** Baseline characteristics (demographics, comorbidities, symptoms, echocardiograph parameters) of patients

	KCH			GSTT		
	HFpEF (*n* = 2220)		Non-HF Dyspnoea	HFpEF (*n* = 3349)		Non-HF Dyspnoea
	Confirmed HFpEF	HFpEF – ESC Criteria	Control	Confirmed HFpEF	HFpEF – ESC Criteria	Control
*n*	231	1992	950	430	2919	2034
Age, mean (SD)	78.8 (10.3)	71.1 (15.4)	57.5 (15.7)	78.1 (10.3)	70.7 (15.1)	56.5 (15.5)
Sex, *n* (%) Female	153 (66.2)	1178 (59.1)	438 (46.1)	257 (59.8)	1561 (53.5)	1080 (53.1)
Ethnicity, *n* (%) White	122 (52.8)	1139 (57.2)	531 (55.9)	249 (57.9)	1701 (58.3)	1170 (57.5)
Black	65 (28.1)	513 (25.8)	196 (20.6)	85 (19.8)	485 (16.6)	350 (17.2)
Asian	17 (7.4)	117 (5.9)	57 (6.0)	29 (6.7)	99 (3.4)	138 (6.8)
Diabetes mellitus type 1, *n* (%)	26 (11.3)	172 (8.6)	30 (3.2)	49 (11.4)	214 (7.3)	123 (6.0)
Diabetes mellitus type 2, *n* (%)	85 (36.8)	494 (24.8)	118 (12.4)	230 (53.5)	962 (33.0)	449 (22.1)
Ischaemic heart disease, *n* (%)	93 (40.3)	455 (22.8)	242 (25.5)	152 (35.3)	843 (28.9)	378 (18.6)
Myocardial infarction, *n* (%)	36 (15.6)	147 (7.4)	95 (10.0)	72 (16.7)	409 (14.0)	205 (10.1)
Cerebrovascular accident, *n* (%)	81 (35.1)	379 (19.0)	95 (10.0)	38 (8.8)	113 (3.9)	61 (3.0)
Hypertensive disorder, *n* (%)	214 (92.6)	1224 (61.4)	423 (44.5)	379 (88.1)	1932 (66.2)	998 (49.1)
Transient ischaemic attack, *n* (%)	47 (20.3)	200 (10.0)	35 (3.7)	40 (9.3)	194 (6.6)	94 (4.6)
Atrial fibrillation, *n* (%)	119 (51.5)	409 (20.5)	60 (6.3)	247 (57.4)	1151 (39.4)	202 (9.9)
Pulmonary hypertension, *n* (%)	40 (17.3)	180 (9.0)	11 (1.2)	51 (11.9)	281 (9.6)	61 (3.0)
Kidney disease, *n* (%)	132 (57.1)	602 (30.2)	156 (16.4)	250 (58.1)	1184 (40.6)	783 (38.5)
Angina, *n* (%)	58 (25.1)	221 (11.1)	101 (10.6)	55 (12.8)	243 (8.3)	104 (5.1)
Dyspnoea, *n* (%)	212 (91.8)	1171 (58.8)	950 (100.0)	353 (82.1)	1784 (61.1)	2034 (100.0)
Dyspnoea at rest, *n* (%)	10 (4.3)	22 (1.1)	NA	34 (7.9)	18 (0.6)	3 (0.1)
Chest pain, *n* (%)	141 (61.0)	652 (32.7)	413 (43.5)	171 (39.8)	787 (27.0)	643 (31.6)
Dizziness, *n* (%)	86 (37.2)	347 (17.4)	125 (13.2)	143 (33.3)	558 (19.1)	341 (16.8)
Presyncope, *n* (%)	5 (2.2)	27 (1.4)	8 (0.8)	8 (1.9)	44 (1.5)	23 (1.1)
Syncope, *n* (%)	33 (14.3)	120 (6.0)	49 (5.2)	49 (11.4)	209 (7.2)	156 (7.7)
NTproBNP (pg/mL), mean (SD)	2714.3 (4412.3)	3086.0 (5519.3)	111.4 (83.5)	3938.9 (7518.0)	3533.3 (6905.5)	122.6 (97.9)
BMI, mean (SD)	30.6 (6.7)	29.2 (7.0)	28.0 (6.1)	31.3 (5.7)	28.8 (5.7)	28.0 (5.3)
IVSd (mm), mean (SD)	1.2 (0.2)	1.2 (0.3)	1.1 (0.2)	1.2 (0.3)	1.2 (0.3)	1.1 (0.3)
LVEDD (mm), mean (SD)	4.5 (0.6)	4.4 (0.6)	4.5 (0.6)	4.5 (0.7)	4.5 (0.7)	4.5 (0.6)
LVEF (%), mean (SD)	59.8 (5.8)	60.5 (6.1)	60.1 (6.3)	59.7 (5.4)	59.3 (5.6)	60.6 (4.9)
LVPWD (mm), mean (SD)	1.1 (0.2)	1.1 (0.2)	1.0 (0.2)	1.1 (0.2)	1.1 (0.2)	1.0 (0.2)
E/e’, mean (SD)	13.7 (6.0)	11.6 (5.9)	8.5 (3.3)	13.0 (6.1)	11.1 (5.3)	8.1 (3.8)
LA volume (ml), mean (SD)	80.2 (34.3)	69.0 (38.4)	50.7 (19.3)	70.2 (40.1)	66.7 (38.9)	44.5 (24.0)
LA volume indexed (mL/m^2^), mean (SD)	41.7 (14.7)	35.7 (17.8)	27.1 (8.9)	42.8 (22.0)	36.7 (16.1)	24.0 (12.5)
LV mass (g), mean (SD)	191.4 (59.6)	185.5 (66.6)	167.2 (55.8)	188.7 (61.1)	190.1 (65.4)	163.6 (59.6)
LV mass indexed (g/m^2^), mean (SD)	101.3 (28.4)	95.8 (33.4)	84.1 (24.2)	104.0 (31.5)	100.7 (29.7)	86.1 (27.9)
PASP (mmHg), mean (SD)	31.6 (12.4)	32.5 (14.3)	23.2 (8.1)	33.1 (13.7)	32.0 (15.3)	23.5 (9.9)
TR max vel (cm/s), mean (SD)	276.1 (52.3)	278.6 (60.2)	237.3 (40.5)	273.7 (55.5)	267.3 (64.9)	227.0 (50.2)
H2FPEF, mean (SD)	5.8 (1.8)	4.3 (2.1)	2.2 (1.6)	4.5 (1.9)	3.5 (2.0)	2.2 (1.7)
IMD, mean (SD)	4.4 (2.2)	4.4 (2.3)	4.7 (2.4)	3.9 (1.9)	4.6 (2.4)	4.7 (2.4)

We randomly divided the KCH patients into 80% for training (a total of 2538 patients: 177 Confirmed, 1601 ESC Criteria, and 760 Control) and 20% for testing (a total of 635 patients: 54 Confirmed, 391 ESC Criteria, and 190 Control), stratified by HFpEF and non-HF groups. The GSTT patients were used for external validation.

Among the four machine learning models: LR, RF, SVM, and XGBoost (XGB), the XGB model achieved the highest performance, with an AUC-ROC of 0.8910 (95% CI, 0.8665–0.9154) in the KCH testing cohort, outperforming the other models (LR:0.8204 [95% CI, 0.7868–0.8540], RF: 0.8472 [95% CI, 0.8161–0.8784], SVM:0.8386 [95% CI, 0.8063–0.8709]). Therefore, we focused on the XGBoost model for further analysis and validation. The full report of the performances of all the models is shown in Supplementary material online, *Table S2*.

**Figure 2:**
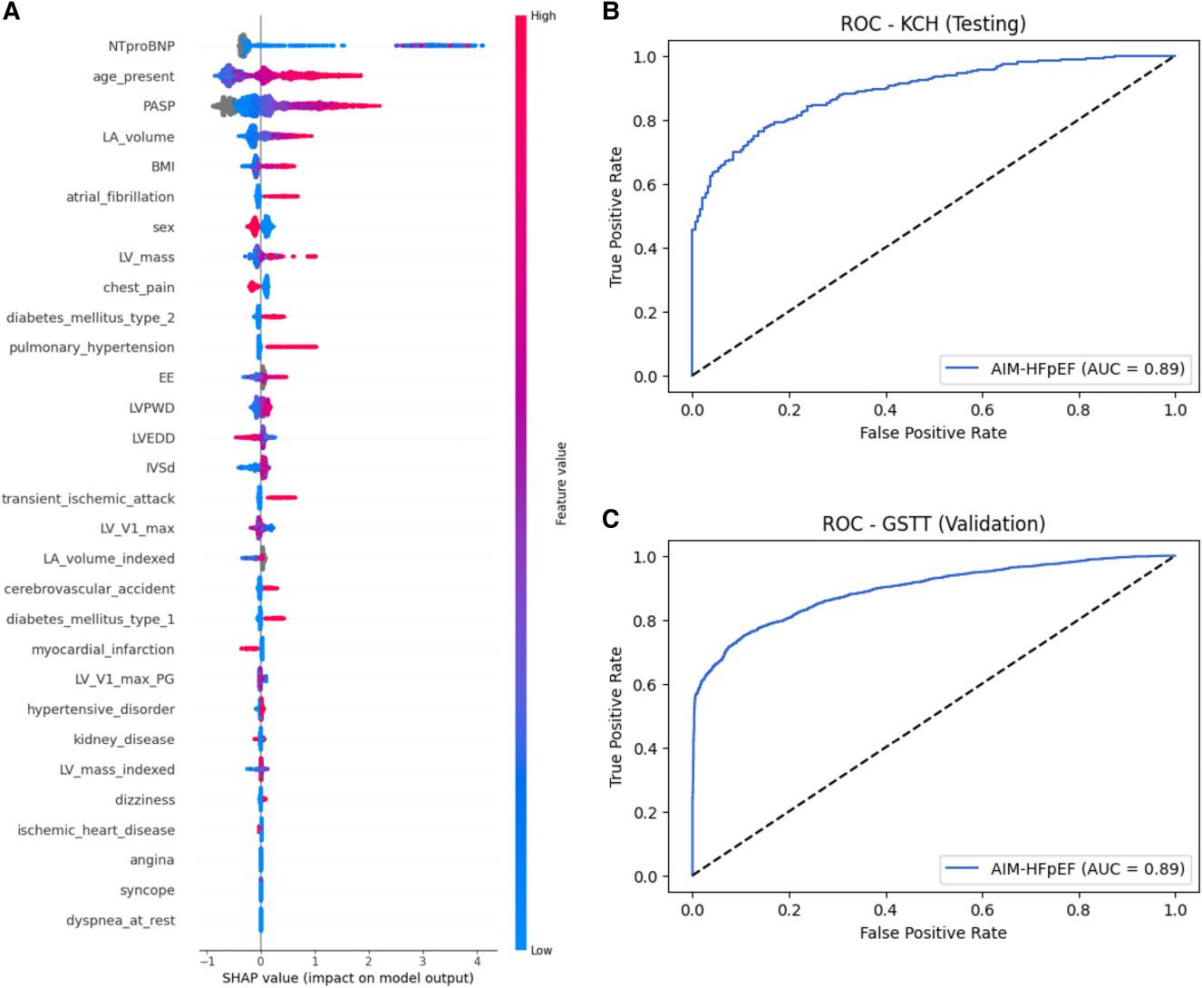
(*A*) SHAP graph for the XGB AIM-HFpEF full model, the *y*-axis is the list of features ranked by their importance with the most important feature at the top. The *x*-axis shows the SHAP values, which indicate the impact of each feature on the model’s prediction. Positive SHAP values show to a tendency of positive HFpEF prediction. The colour reflects the values of the features (for continuous features: red represents higher values and blue lower values; for sex: red represents male and blue female; for binary features like comorbidities and symptoms: red represents positive and blue negative). (*B*) ROC curve of KCH testing cohort. (*C*) ROC curve of GSTT validation cohort.

We employed SHAP analysis to understand the contribution of individual features to the model’s predictions from XGB as shown in *Figure 2A*. The top contributing features include NTproBNP, age, PASP, LA volume, and BMI. Age, PASP, and BMI are also included in the H2FPEF formula but the XGB model has no prior information of this. The SHAP values of the top features are shown in Supplementary material online, *Table S3*. *Figure 2B* and *C* shows the receiver operating characteristic (ROC) curves for the XGB prediction model on the KCH testing dataset and GSTT validation dataset, respectively. The AUC for the GSTT validation dataset was 0.8934 (95% CI, 0.8852–0.9016) for the XGB model.

### Simplified model

We selected the top 10 features from the SHAP analysis for a simplified model. The choice of 10 features was to balance between model simplicity and performance. Including a smaller number of features while not decreasing the performance significantly is crucial for the simplified model to remain practical, particularly where manual data input is required in certain scenarios. The 10 features are as follows: NTproBNP, age, PASP, LA volume, BMI, AF, sex, LV mass, chest pain, and diabetes mellitus.

A simplified version of the machine learning models was next built using the 10 features. The XGB model again performed the best (AUC-ROC of 0.8801 [95% CI, 0.8539–0.9063]) among the four models (LR: 0.7962 [95% CI, 0.7603–0.8321], RF: 0.8202 [95% CI, 0.7851–0.8553], SVM: 0.8181 [95% CI, 0.7835–0.8527]) for the KCH testing cohort. The AUC of the simplified model is slightly less than that in the full model (AUC of 0.8900 [95% CI, 0.8817-0.8983] for the GSTT validation cohort), but it is more usable in practice. The individual performances of the model on the HFpEF – Confirmed and HFpEF – ESC Criteria groups are shown in Supplementary material online, *Figure S2*. The calibration curves for the two HFpEF groups in the GSTT validation cohort is shown in Supplementary material online, *Figure S3*. The simplified XGB model is used for subsequent comparison and subgroup analysis. *Table 2* shows the sensitivity, specificity, positive predictive value (PPV), and negative predictive value (NPV) of the main results.

**Table 2:** Sensitivity, specificity, positive predictive value (PPV), and negative predictive value (NPV) of the main results

Cohort	Model	Sensitivity	Specificity	PPV	NPV
KCH testing cohort	AIM-HFpEF	0.8694	0.6316	0.8465	0.6742
	H2FPEF	0.6575	0.7895	0.9231	0.3750
	HFpEF-ABA	0.7703	0.5839	0.8230	0.5031
GSTT validation cohort	AIM-HFpEF	0.8901	0.6160	0.7924	0.7730
	H2FPEF	0.6675	0.7500	0.6692	0.7486
	HFpEF-ABA	0.7977	0.5649	0.5687	0.7952

### Comparison with H2FPEF and HFpEF-ABA scores

**Figure 3:**
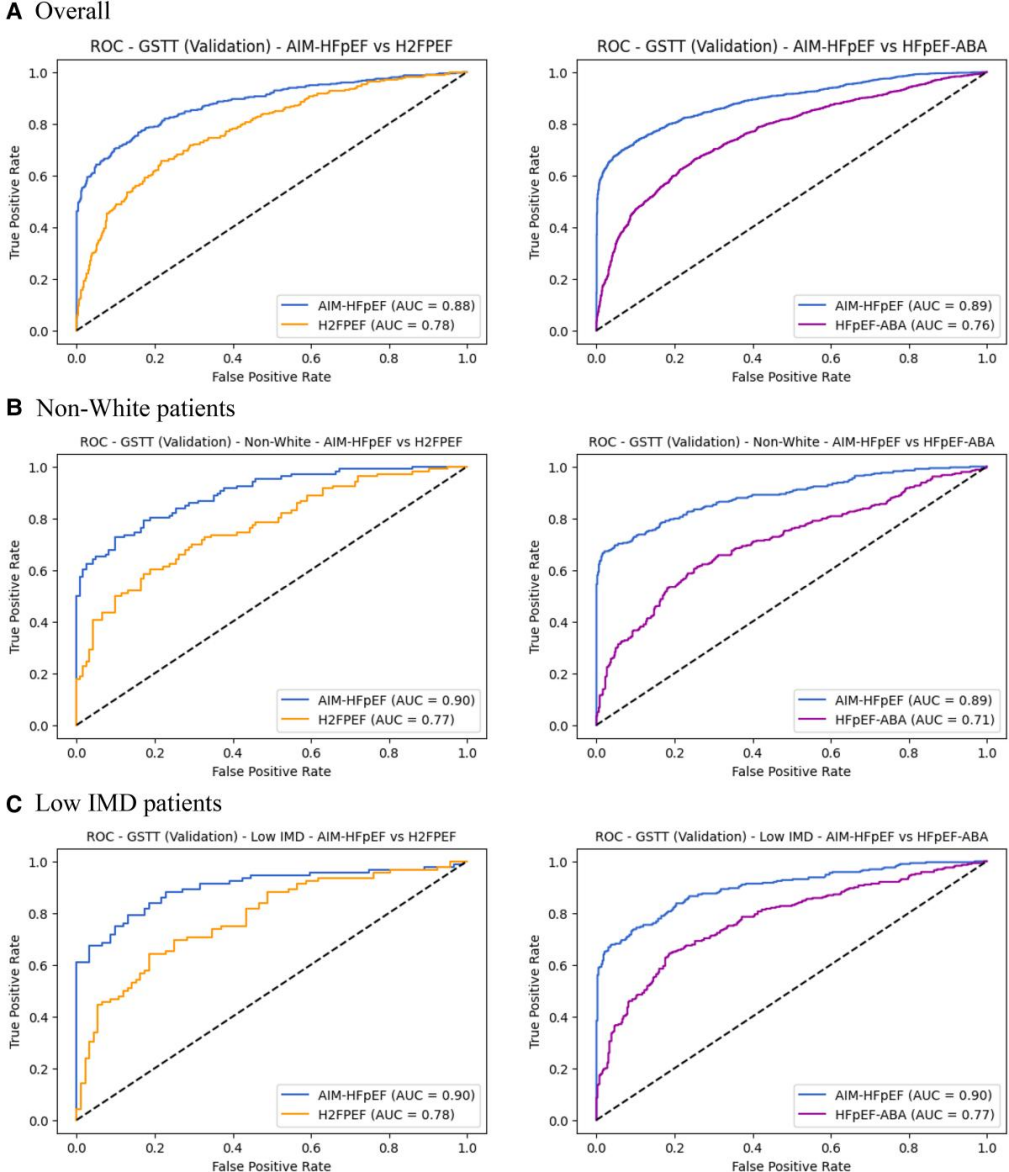
Comparisons of simplified XGB, H2FPEF, and HFpEF-ABA in the GSTT validation cohort. (*A*) Overall cohort. (*B*) Non-White patients. (*C*) Low IMD patients.

To evaluate the performance of the simplified XGB model in comparison with established clinical scoring systems, we compared it to the widely used H2FPEF score and the more recently published HFpEF-ABA score. In the KCH testing cohort (*n* = 635), 182 (29%) patients had complete data for all five variables (age, BMI, PASP, E/e’, and AF) required to calculate the H2FPEF probability (AUC, AIM-HFpEF: 0.8610 [95% CI, 0.8040–0.9181], H2FPEF: 0.7873 [95% CI, 0.7075–0.8672], *P* = 0.0136), while 483 (76%) patients had all three variables (age, BMI, and AF) necessary for the HFpEF-ABA score calculation (AUC, AIM-HFpEF: 0.8729 [95% CI, 0.8420–0.9038], HFpEF-ABA: 0.7425 [95% CI, 0.6949–0.7901], *P* < 0.0001). In the GSTT validation cohort (*n* = 5383), 914 (17%) patients had the full set of variables to compute the H2FPEF probability (AUC, AIM-HFpEF: 0.8808 [95% CI, 0.8578–0.9038]), H2FPEF: 0.7805 [95% CI, 0.7505–0.8105], *P* < 0.0001) and 3497 (65%) patients had the variables required for the HFpEF-ABA score (AUC, AIM-HFpEF: 0.8880 [95% CI, 0.8765–0.8996], HFpEF-ABA: 0.7624 [95% CI, 0.7462–0.7787], *P* < 0.0001). *Figure 3A* presents the ROC curves comparing the performance of the simplified XGB model, H2FPEF score, and HFpEF-ABA score in the GSTT validation cohort. Comparison for the KCH testing cohort is shown in Supplementary material online, *Figure S4*(*A*). *Table 2* shows the sensitivity, specificity, PPV, and NPV of the main results.

### Subgroup analysis

In GSTT validation cohort (*n* = 5383), there were 1186 (22%) non-White patients and 1091 (20%) patients from the lowest quintile of IMD.

Non-White patients: In the GSTT validation cohort, the number of patients included in the comparison for the H2FPEF and HFpEF-ABA scores were 228 (AUC, AIM-HFpEF: 0.8958 [95% CI, 0.8559–0.9357], H2FPEF: 0.7681 [95% CI, 0.7071–0.8291], *P* < 0.0001) and 827 (AUC, AIM-HFpEF: 0.8873 [95% CI, 0.8630–0.9116], HFpEF-ABA: 0.7101 [95% CI, 0.6734–0.7468], *P* < 0.0001), respectively. *Figure 3B* shows the ROC curves for the GSTT validation cohort. Comparisons for the KCH testing cohort is shown in Supplementary material online, *Figure S4*(*B*).

Low IMD patients: In the GSTT validation cohort, the corresponding numbers were 184 (AUC, AIM-HFpEF: 0.8990 [95% CI, 0.8519–0.9460], H2FPEF: 0.7793 [95% CI, 0.7127–0.8459], *P* < 0.0001) and 681 (AUC, AIM-HFpEF: 0.8981 [95% CI, 0.8735–0.9227], HFpEF-ABA: 0.7745 [95% CI, 0.7383–0.8107], *P* < 0.0001), respectively. *Figure 3C* shows the ROC curves for the GSTT validation cohort. Comparisons for the KCH testing cohort is shown in Supplementary material online, *Figure S4*(*C*).

To understand which patients the model identifies that current clinical practice misses, a comparative analysis of H2FPEF diagnostic features between the HFpEF – Confirmed and HFpEF – ESC Criteria groups is performed. As shown in Supplementary material online, *Figure S6*, patients in the ESC Criteria group were less likely to have AF, high BMI, or be over age 60. These are factors known to influence clinical recognition of HFpEF. This shows the value of including under-documented cases in training and highlights the model’s potential to reduce diagnostic disparities.

### Outcome analysis

**Figure 4:**
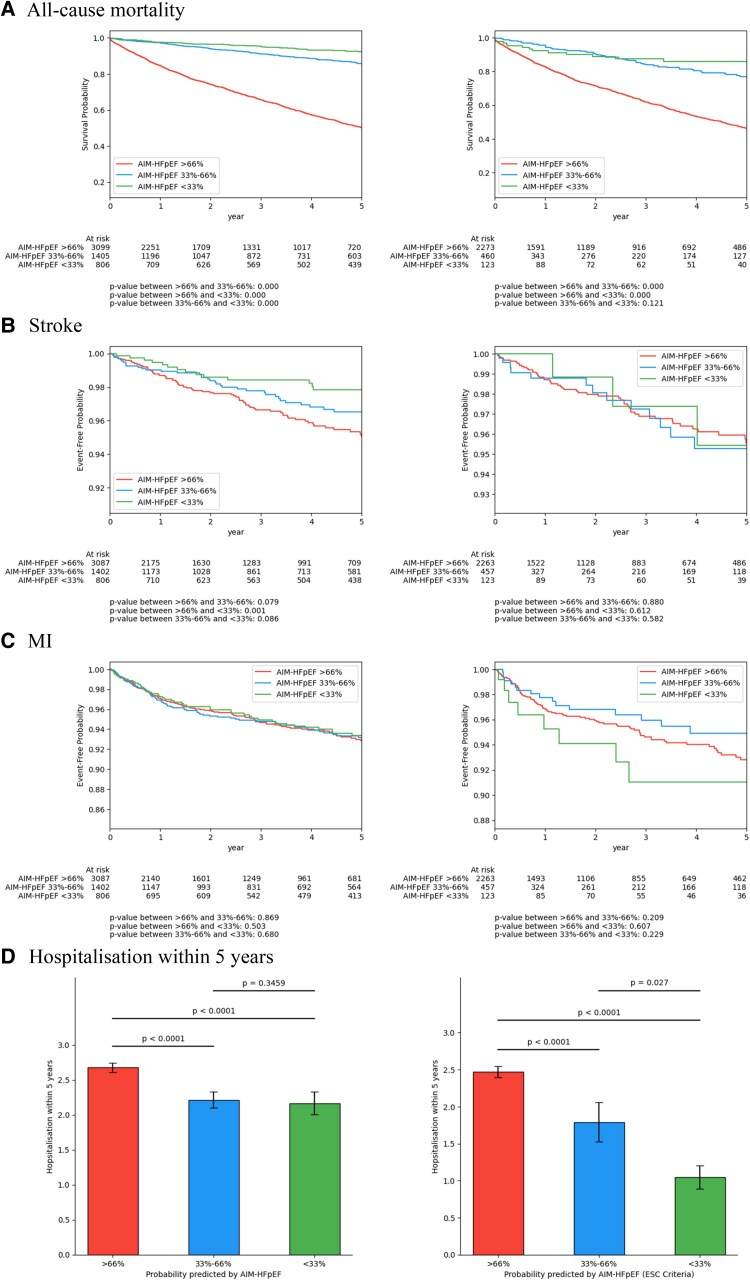
Outcome of GSTT validation cohort divided into three groups based on AIM-HFpEF predicted probability tertiles (left: overall cohort, right: HFpEF – ESC criteria cohort). (*A*) All-cause mortality. (*B*) Stroke. (*C*) MI. (*D*) Hospitalization within 5 years.

In the outcome analysis, we selected patients with a predicted probability ≥90% of having HFpEF from the models and investigated their all-cause mortality over a 5 year period. In the KCH testing cohort, the AIM-HFpEF model identified 223 patients and 87 (39%) of them died within 5 years of HF diagnosis. H2FPEF identified 39 patients and 13 (33%) died within 5 years, while HFpEF-ABA identified 87 and 29 (33%) of them died. In the GSTT validation cohort, the numbers are AIM-HFpEF: 2318 and 961 (41%); H2FPEF: 105 and 53 (50%); HFpEF-ABA: 606 and 193 (32%). Kaplan–Meier (KM) curves of the outcome analysis for all-cause mortality, MI, and stroke are shown in Supplementary material online, *Figure S5*. We also investigated whether the AIM-HFpEF model can predict outcome by dividing the GSTT validation cohort into three groups based on AIM-HFpEF predicted probability tertiles in *Figure 4*. In the overall cohort, AIM-HFpEF produced probabilities that were associated with an increased risk of death (*P* < 0.0001), stroke (*P* = 0.001) and hospitalization (*P* < 0.0001) when comparing the highest tertile to the middle tertile. In the HFpEF – ESC Criteria cohort, AIM-HFpEF probabilities were associated with an increased risk of death (*P* < 0.0001) and hospitalization (*P* < 0.0001).

## Discussion

Using well-validated NLP and machine learning techniques applied to routinely collected data from the EHR, we have developed the AIM-HFpEF predictive model to accurately detect patients with HFpEF. Importantly from a health equity point of view and to address algorithmic bias,^21^ it performs well in patients of non-White ethnicity and in patients from areas of higher socioeconomic deprivation.

AIM-HFpEF is designed to be fully automated and integrated into EHR platforms, has been externally validated, and performs favourably to current diagnostic and screening models. It is anticipated that patients with a high likelihood of HFpEF as ascertained by the AIM-HFpEF model could be identified to the clinician by way of an electronic pop-up prompt within the EHR, with subsequent referral to a cardiologist for specialist assessment and initiation of treatment if appropriate.

In constructing AIM-HFpEF, we have taken the novel step of including data from patients who met ESC diagnostic criteria for HFpEF but did not have a documented diagnosis in their clinical records. We see this as a potential key approach in addressing the issue of underdiagnosis in HFpEF. Conceptually, it can be considered that the characteristics of the HFpEF – ESC Criteria patients are the clinically most important predictors, not captured through analysis solely of diagnosed HFpEF. One possible reason is that these patients not yet diagnosed have information missing in their structured data and only present in unstructured form. Patients within the HFpEF – Confirmed group have *already been diagnosed* and therefore are in less of a need of a predictive model, whereas the patients within the HFpEF – ESC Criteria group are those who are being missed by current diagnostic methods.

The requirement for an echocardiogram within 1 year of the documented clinical event (HF or dyspnoea mention) was chosen to balance between temporal accuracy and practical feasibility. We selected a 1 year window as a pragmatic compromise: it maintains reasonable temporal proximity to the clinical presentation while ensuring that the dataset remains sufficiently large and representative of real-world clinical practice.

A key concern of AI-based disease prediction tools is the risk of potentiating any biases contained within the training dataset. A key aim of this study was to ensure that AIM-HFpEF retained good performance in diagnosing the significant minority of HFpEF patients of non-White race and ethnicity, to ensure alignment with future frameworks for assessing algorithmic bias.^21^ We note that the derivation and validation populations of the HFPEF-ABA score were overwhelming of White ethnicity and therefore its generalizability to non-White populations has not previously been ascertained. Furthermore, several studies have identified a lack of generalizability in the H2FPEF score *viz-a-viz* its performance in non-White populations, likely at least in part due to the heavy weighting afforded to a diagnosis of AF in the H2FPEF score (only a small proportion of Black patients with HFpEF have a diagnosis of AF).^8^ Our results show that AIM-HFpEF performs better than H2FpEF and HFpEF-ABA in non-White patients in the UK, although we do note that both existing scores have reasonable performance in this patient group and our study can also be considered as additional external validation of these models.

The predictors identified in the full model can be related to HFpEF either in terms of direct pathophysiological mechanisms or by their relation to clinical features associated with the syndrome. In contrast to HFpEF-ABA, in our SHAP analysis we found several echocardiogram variables to be significant predictors of HFpEF and therefore our model includes a number of echocardiogram measures, i.e. PASP, left atrial volume, and left ventricle mass. Among the most significant variables (i.e. NTproBNP, age, PASP, LA volume, BMI, AF, sex, LV mass, chest pain, and diabetes mellitus), NTproBNP, PASP, LA volume, and LV mass are specified in the ESC guidelines for diagnosing HFpEF.^17^ It is well established that the prevalence of HFpEF increases with advancing age,^22^ and there are studies between BMI and HFpEF.^23^ Furthermore, women are found to be overrepresented in HFpEF.^24^ As expected, there is significant overlap between the variables included in AIM-HFpEF and those in other HFpEF predictive models. A key difference is the inclusion of NTproBNP in our model. Given that not all patients will have NTproBNP results available, we have confirmed the acceptable model performance even when natriuretic peptide results are not available (see Supplementary Document  *S2*).

### Strengths and limitations

A key strength of an EHR-based approach is that it lends itself to automation, i.e. there is no requirement for an a priori suspicion of HFpEF as is the case with diagnostic scores such as H2FPEF and HFA-PEFF. Conversely, the use of routinely collected, retrospective data does have key limitations, including non-standardized reporting of clinical features across the two participating centres, therefore requiring prospective validation.

As discussed above, the inclusion of the HFpEF – ESC Criteria patients in both the derivation and validation datasets is a strength of our approach, potentially allowing the final model to be much more generalizable and less prone to bias compared with diagnostic models derived from smaller, more highly preselected groups of HFpEF – Confirmed patients.

Another limitation of our model is the reliance on an advanced data extraction platform to employ NLP methods and retrieve clinical data from the EHR. Hospital systems with informatics capabilities to employ our model are in the minority globally, particularly in low- and lower-middle income countries, despite the CogStack technology being low cost and light weight, and available open source. We have sought to mitigate this limitation by producing an alternative prediction model that does not require advanced EHR data analytic capabilities. This **Simplified Model** could potentially be accessed via a smartphone app to enable clinicians to define the likelihood of a HFpEF diagnosis.

While natriuretic peptide testing is commonly used in the evaluation of unexplained dyspnoea 24, the inclusion of NTproBNP as a predictive feature in the model may introduce confounding as it reflects some degree of clinician suspicion for cardiac pathology. To evaluate this, we conducted a sensitivity analysis excluding NTproBNP, which confirmed that the AIM-HFpEF model maintained performance in the absence of this feature (KCH testing cohort: all features AUROC = 0.8910, without NTproBNP AUROC = 0.8656, GSTT validation cohort: all features AUROC = 0.8934, without NTproBNP AUROC = 0.8118, Supplementary Document  *S2*).

A further limitation is that although both the derivation and validation cohorts come from separate large multi-hospital NHS trusts, they are both within the same large urban metropolis, i.e. London. Further external validation in different settings is therefore required to ensure generalizability of our findings across broader geographic areas. Further work will therefore involve assessment of wider generalizability both in larger UK datasets and in international datasets. Additional future avenues including prospective validation will be a key step towards assessing the ability of AIM-HFpEF to affect patient outcomes through improved diagnosis via the model. Incorporation of primary care data will be important to ensure accurate diagnosis in the unknown proportion of HFpEF – ESC Criteria patients without clinical data within secondary care. We acknowledge that reliance on echocardiographic data may limit the model’s utility in some resource-limited settings. While our model is designed for automation in sites that can routinely collect these parameters in the EHRs, we recognize the importance of creating tools for diverse healthcare settings. Future work could explore the development of alternative models that rely on more accessible parameters without compromising diagnostic accuracy.

### Future implementation and clinical integration

Future implementation science will include the prospective evaluation of AIM-HFpEF deployed within the EHR systems of multiple hospital networks, leveraging the CogStack platform^19^ to enable real-time feedback loops influencing patient care. This will include the following:

1. Technical validation of score thresholds and alerting mechanisms;
2. Clinical validation with follow-up of outcomes including mortality, stroke, and specialist referral;
3. Evaluation of clinician engagement modes (e.g. active alerts, passive nudges, dashboard integration).

Our implementation strategy will incorporate principles of fairness, usability, transparency, and equity, in line with the FUTURE-AI guidelines for trustworthy AI in healthcare.^25^ In particular, we aim to ensure the model’s performance remains consistent across diverse populations and to design clinician interfaces that minimize alert fatigue while supporting timely decision-making.

## Conclusion

In this study we describe the use of AI methods to develop an automated, EHR-based diagnostic prediction model for HFpEF. The AIM-HFpEF model has been externally validated and is seen to perform favourably to existing diagnostic and screening models and is accurate in non-White patients and in those from areas of high socioeconomic deprivation. However, additional validation in other geographic and healthcare settings will be needed to confirm broader generalizability.

## Supplementary Material

ztaf107_Supplementary_Data

## Data Availability

The data and code underlying this article will be shared on reasonable request to the corresponding author.
