# Supplementary material for: Artificial intelligence methods to detect heart failure with preserved ejection fraction within electronic health records: an equitable disease detection model
Source: Eur Heart J Digit Health. 2025 Sep 16;7(1):ztaf107. doi: 10.1093/ehjdh/ztaf107 (PMC12821069; doi:10.1093/ehjdh/ztaf107)
Supplement: ztaf107_Supplementary_Data [file ztaf107_supplementary_data.zip › Supplementary_Document_1.docx]

**Supplementary Document 1. The NLP pipeline used in the study**

To handle the large volume of patient data at King’s College Hospital NHS Foundation Trust (KCH) and Guy’s and St Thomas’ NHS Foundation Trust (GSTT), CogStack, a centralised and open-sourced data platform, is deployed at each of the sites to aggregate both structured and unstructured data from electronic health records (EHRs). Structured data includes patients’ demographic details (date of birth, sex, ethnicity) and laboratory results, whereas unstructured content includes free-text documents such as clinical notes, referral letters, and discharge summaries. By providing a single interface for searching across patient records, CogStack simplifies information retrieval and enhances data accessibility at each site.

Given the large amount of text-based content in the EHR, we used MedCAT to identify and label key medical concepts in the unstructured documents. MedCAT uses a combination of machine learning approaches, involving both supervised and unsupervised training, to achieve high accuracy when extracting clinical concepts. Its concept disambiguation strategy involves word-embedding similarity and a Bidirectional Long Short-Term Memory (Bi-LSTM) model for contextual processing of concepts, taking into account contexts like negation, temporality, and who is experiencing the condition (e.g., patient versus non-patient references). Spelling corrections are also automatically applied during this annotation phase. MedCAT also offers flexibility to incorporate large language models (LLMs) like Bidirectional Encoder Representations from Transformers (BERT), leveraging transformer-based architectures to further improve performance in biomedical named entity recognition (NER) and linking tasks.

For our study, MedCAT initially performed unsupervised recognition of concepts drawn from SNOMED-CT. It targeted SNOMED concepts under the parent categories of Clinical Finding, Disorder, Organism, and Event. This unsupervised pass was based on training with the MIMIC-III dataset, and was followed by supervised fine-tuning via MedCATTrainer. The final system assigned meta-annotations, indicating whether the concept was relevant, negated, or referred to a non-patient subject, helping to exclude hypothetical mentions or conditions applicable to someone other than the patient.

We evaluated MedCAT’s performance by comparing its textual annotations with those of a domain expert across more than 5,600 annotations in over 265 clinical documents. Precision, recall, and F1 scores were consistently high (i.e., greater than 0.9), confirming the reliability of MedCAT as our core natural language processing (NLP) tool for detecting and classifying clinical concepts.
