# Supplementary material for: Artificial intelligence methods to detect heart failure with preserved ejection fraction within electronic health records: an equitable disease detection model
Source: Eur Heart J Digit Health. 2025 Sep 16;7(1):ztaf107. doi: 10.1093/ehjdh/ztaf107 (PMC12821069; doi:10.1093/ehjdh/ztaf107)

**Supplementary Figure 1.** CONSORT (Consolidated Standards of Reporting Trails) diagram of patient inclusion and exclusion criteria. KCH = King’s College Hospital NHS Foundation Trust; GSTT = Guy’s and St Thomas’ NHS Foundation Trust; ESC = European Society of Cardiology; HF = heart failure; HFpEF = heart failure with preserved ejection fraction; LVEF = left ventricular ejection fraction.


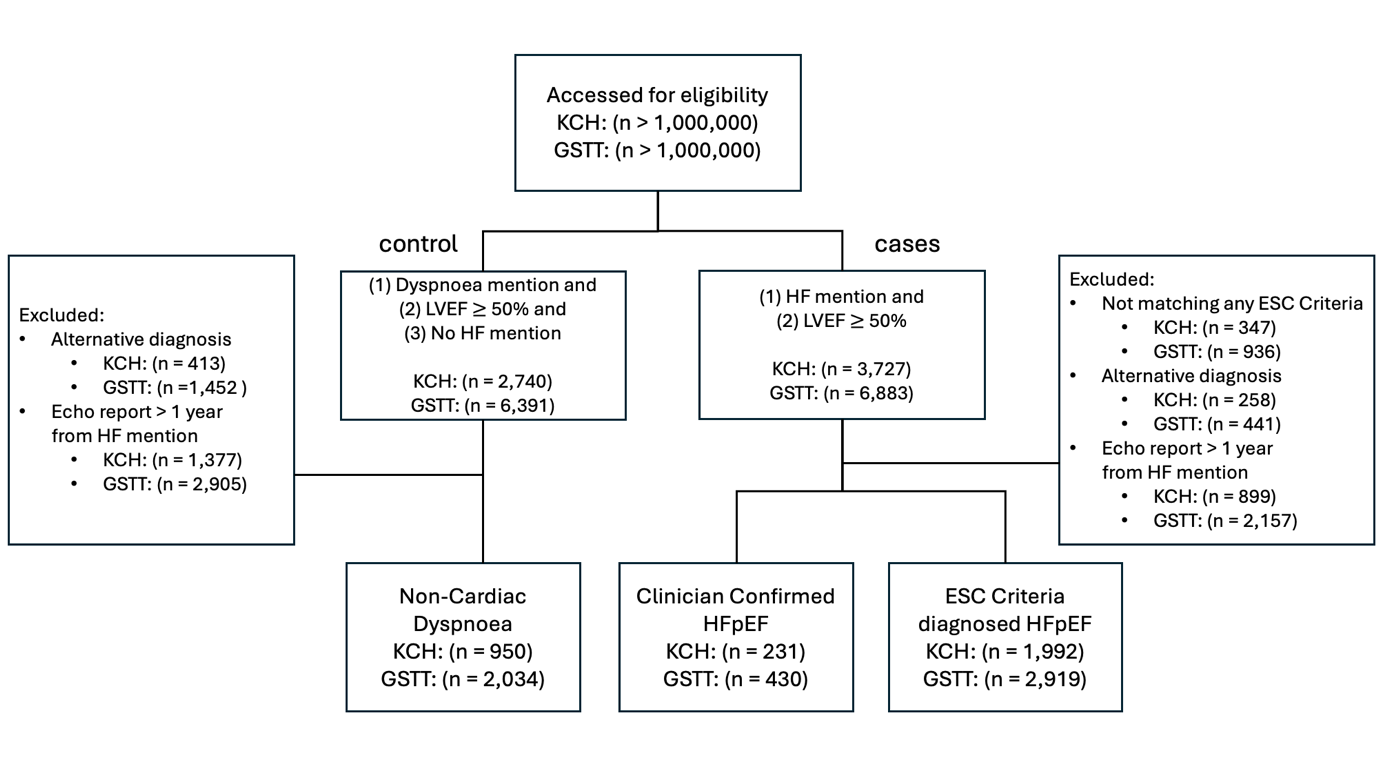

Supplement: ztaf107_Supplementary_Data [file ztaf107_supplementary_data.zip › Supplementary_Figure_1.docx]
