# Supplementary material for: Artificial intelligence methods to detect heart failure with preserved ejection fraction within electronic health records: an equitable disease detection model
Source: Eur Heart J Digit Health. 2025 Sep 16;7(1):ztaf107. doi: 10.1093/ehjdh/ztaf107 (PMC12821069; doi:10.1093/ehjdh/ztaf107)
Supplement: ztaf107_Supplementary_Data [file ztaf107_supplementary_data.zip › Supplementary_Figure_2.docx]

**Supplementary Figure 2.** Performances of the AIM-HFpEF simplified model on HFpEF – Confirmed (left) and HFpEF – ESC Criteria (right) groups. (a) Overall Cohort. (b) Comparison with H2FPEF. (c) Comparison with HFpEF-ABA.

| (a) AIM-HFpEF simplified model on HFpEF – Confirmed patients (AUC: 0.9604 [95% CI, 0.9493-0.9715]) and HFpEF – ESC Criteria patients (AUC: 0.8797 [95% CI, 0.8705-0.8888]) | |
| --- | --- |
| 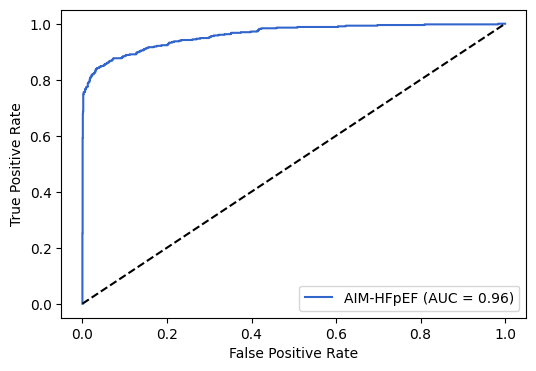 | 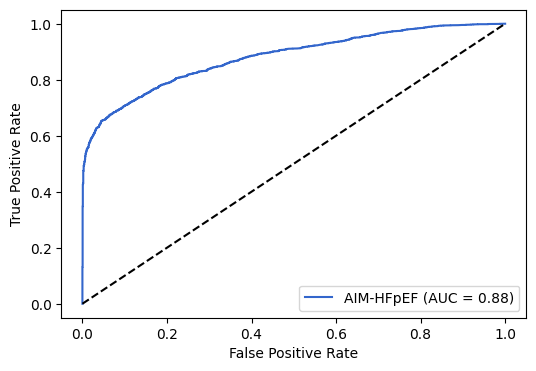 |
| (b) Comparison with H2FPEF in GSTT validation cohort. Left, AIM-HFpEF: 0.9600 [0.9241-0.9958], H2FPEF: 0.8875 [0.8383-0.9368], *P*=0.0030. Right, AIM-HFpEF: 0.8698 [0.8446-0.8950], H2FPEF: 0.7656 [0.7335-0.7978], *P*<0.0001. | |
| 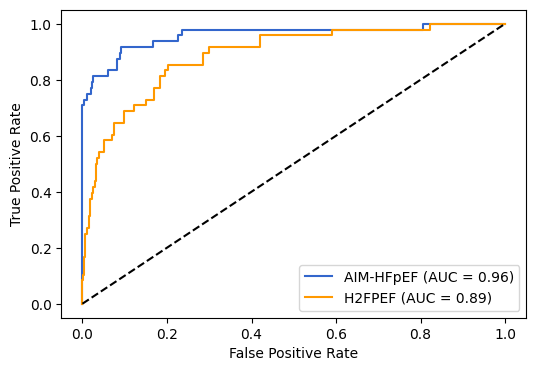 | 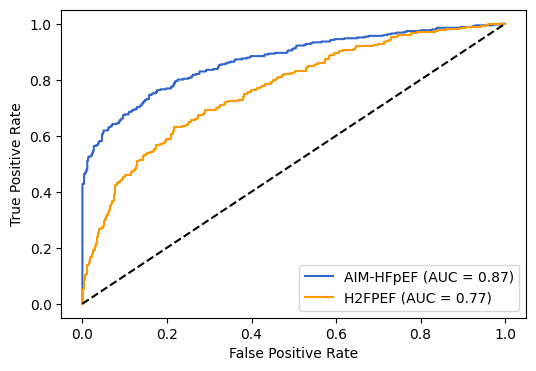 |
| (c) Comparison with HFpEF-ABA in GSTT validation cohort. Left, AIM-HFpEF: 0.9716 [0.9586-0.9846], HFpEF-ABA: 0.8827 [0.8572-0.9082], *P*<0.0001. Right, AIM-HFpEF: 0.8756 [0.8628-0.8885], HFpEF-ABA: 0.7446 [0.7270-0.7622], *P*<0.0001. | |
| 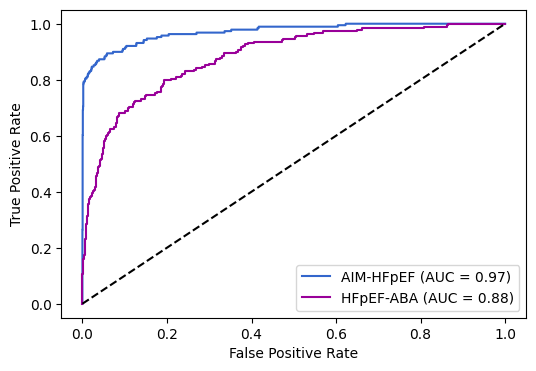 | 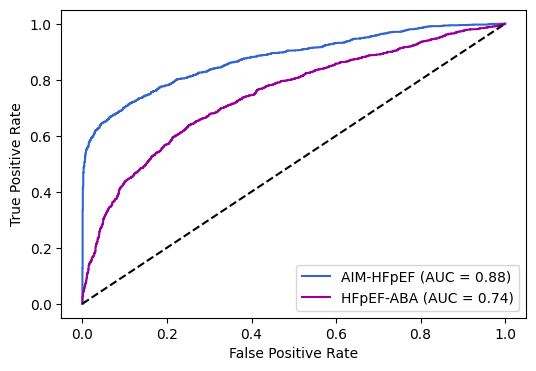 |
