# Supplementary material for: Artificial intelligence methods to detect heart failure with preserved ejection fraction within electronic health records: an equitable disease detection model
Source: Eur Heart J Digit Health. 2025 Sep 16;7(1):ztaf107. doi: 10.1093/ehjdh/ztaf107 (PMC12821069; doi:10.1093/ehjdh/ztaf107)

**Supplementary Figure 3.** Calibration curves of predicted probabilities produced by the AIM-HFpEF simplified model. The HFpEF – ESC Criteria group is more calibrated, while the HFpEF – Confirmed group tends to have over-estimated probabilities when predicted probabilities are low.

|  |
| --- |


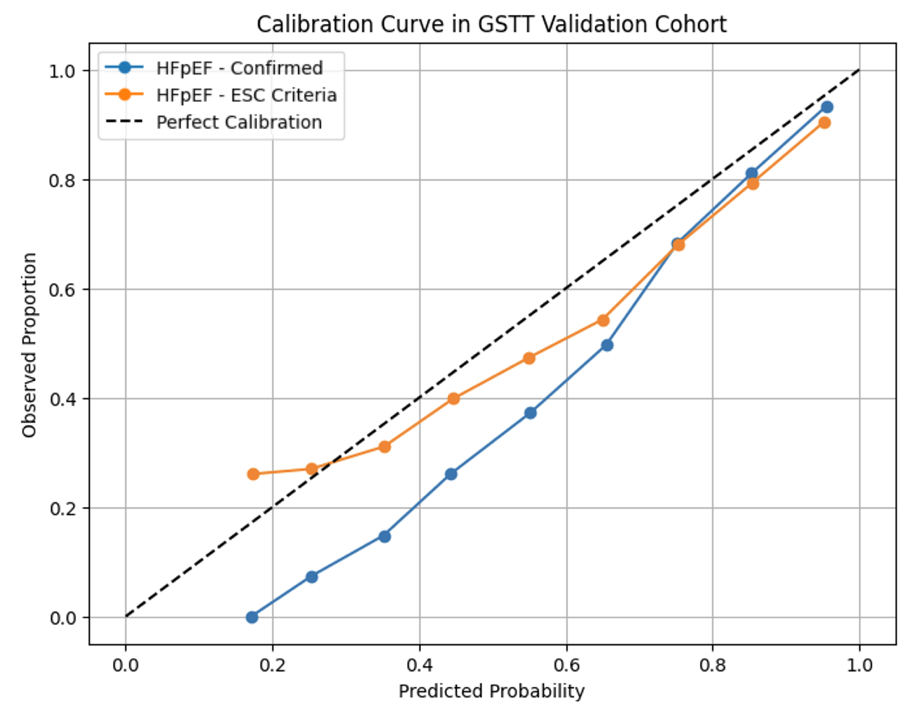

Supplement: ztaf107_Supplementary_Data [file ztaf107_supplementary_data.zip › Supplementary_Figure_3.docx]
