# Supplementary material for: Artificial intelligence methods to detect heart failure with preserved ejection fraction within electronic health records: an equitable disease detection model
Source: Eur Heart J Digit Health. 2025 Sep 16;7(1):ztaf107. doi: 10.1093/ehjdh/ztaf107 (PMC12821069; doi:10.1093/ehjdh/ztaf107)
Supplement: ztaf107_Supplementary_Data [file ztaf107_supplementary_data.zip › Supplementary_Figure_4.docx]

**Supplementary Figure 4.** ROC curves for comparisons with H2FPEF and HFpEF-ABA scores in KCH testing cohort, (a) Overall (b) Non-White patients (c) Low IMD patients.

| (a) KCH testing cohort (Overall) | |
| --- | --- |
| 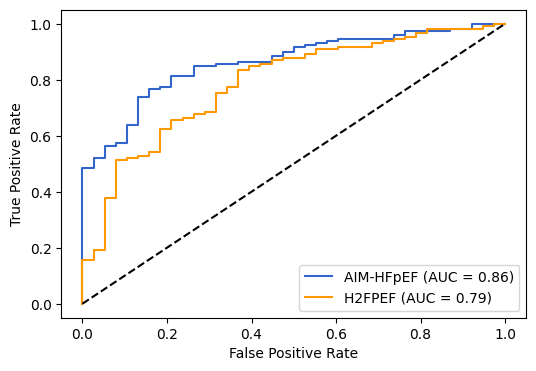 | 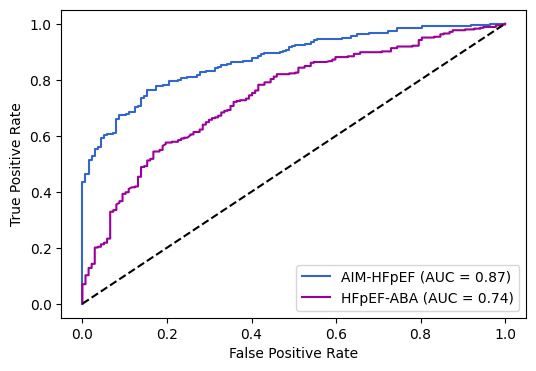 |
| (b) KCH testing cohort (Non-White patients)  In the KCH testing cohort, we compared the performance of the models in 74 non-White patients for the H2FPEF score (AUC, AIM-HFpEF: 0.8675 [95% CI, 0.7739-0.9610], H2FPEF: 0.7899 [95% CI, 0.6774-0.9023], *P*=0.1409) and 159 patients for the HFpEF-ABA score (AUC, AIM-HFpEF: 0.8570 [95% CI, 0.7978-0.9162], HFpEF-ABA: 0.7542 [95% CI, 0.6725-0.8359], *P*=0.0059). | |
| 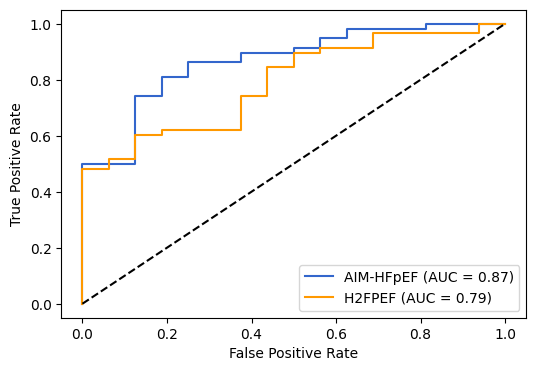 | 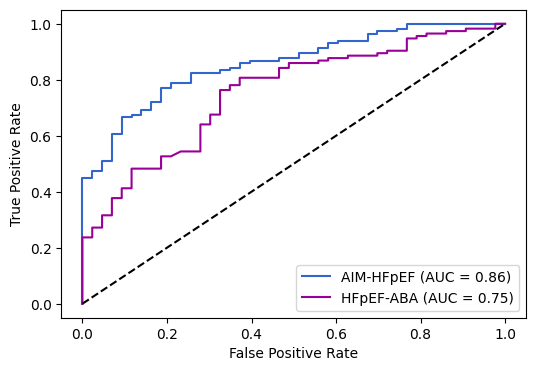 |
| (c) KCH testing cohort (Low IMD patients)  For the low IMD group, in the KCH testing cohort, we compared 44 patients using the H2FPEF score (AUC, AIM-HFpEF: 0.8615 [95% CI, 0.6759-1.0000], H2FPEF: 0.7538 [95% CI, 0.6080-0.8996], *P*=0.3101) and 93 patients using the HFpEF-ABA score (AUC, AIM-HFpEF: 0.8818 [95% CI, 0.8075-0.9560], HFpEF-ABA: 0.7426 [95% CI, 0.6245-0.8607], *P*=0.0107). | |
| 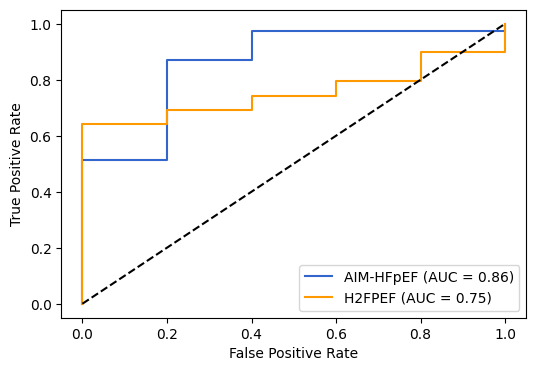 | 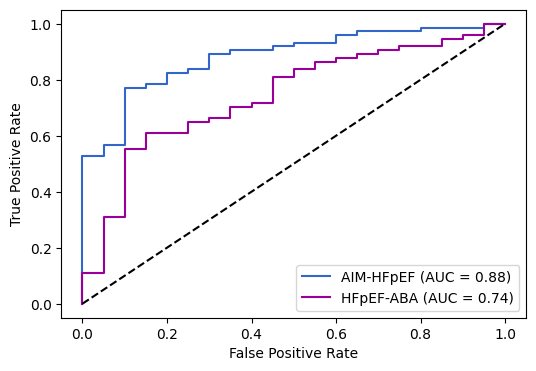 |
