# Supplementary material for: Artificial intelligence methods to detect heart failure with preserved ejection fraction within electronic health records: an equitable disease detection model
Source: Eur Heart J Digit Health. 2025 Sep 16;7(1):ztaf107. doi: 10.1093/ehjdh/ztaf107 (PMC12821069; doi:10.1093/ehjdh/ztaf107)
Supplement: ztaf107_Supplementary_Data [file ztaf107_supplementary_data.zip › Supplementary_Figure_5.docx]

**Supplementary Figure 5.** Kaplan-Meier (KM) curves in KCH testing cohort (left) and GSTT validation cohort (right) for patients having a predicted probability ≥ 90% of having HFpEF by the models with (a) all-cause mortality (b) MI and (c) Stroke within 5 years.

| (a) All-cause mortality within 5 years | |
| --- | --- |
| 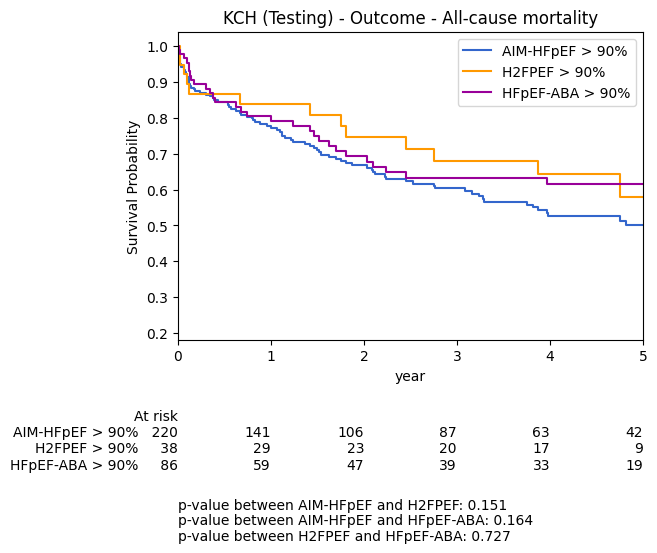 | 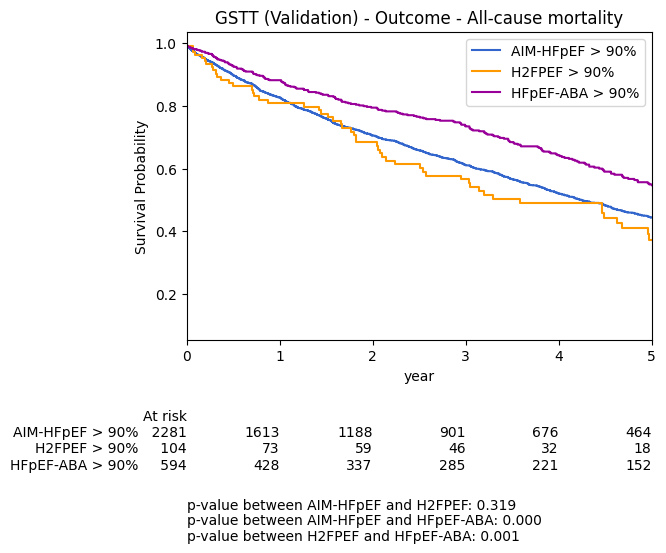 |
| (b) MI within 5 years | |
| 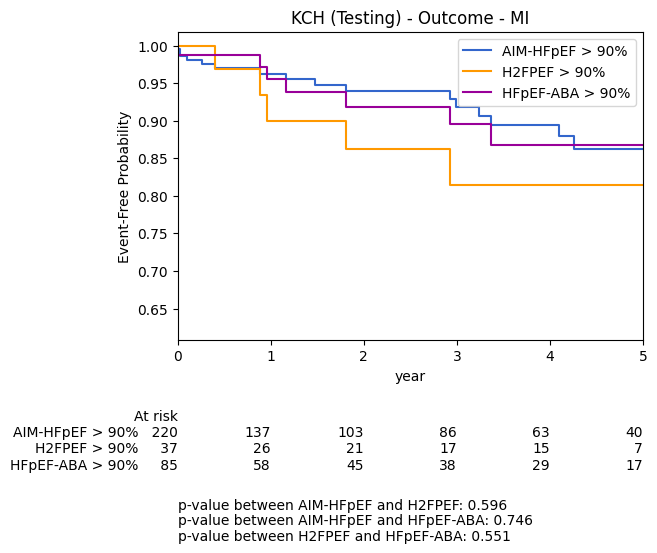 | 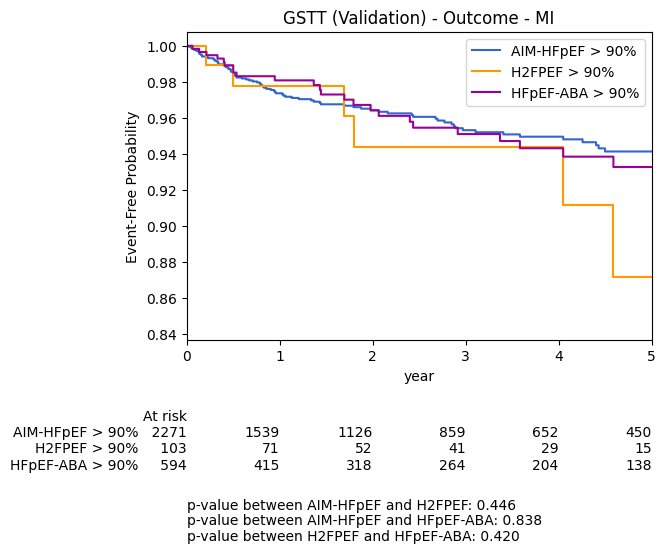 |
| (c) Stroke within 5 years | |
| 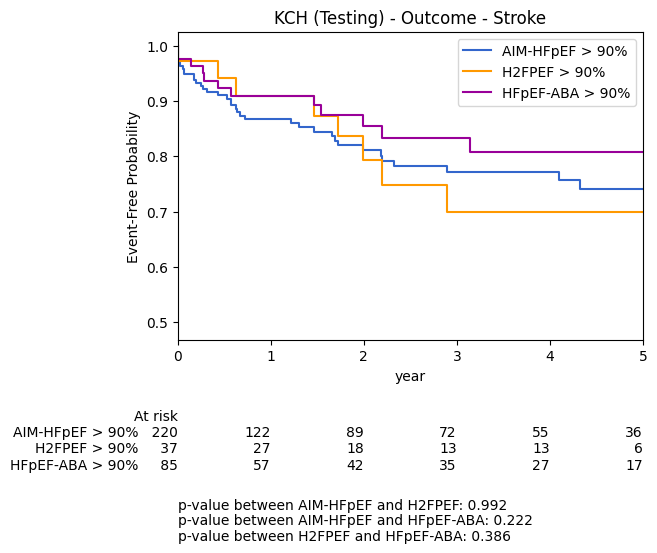 | 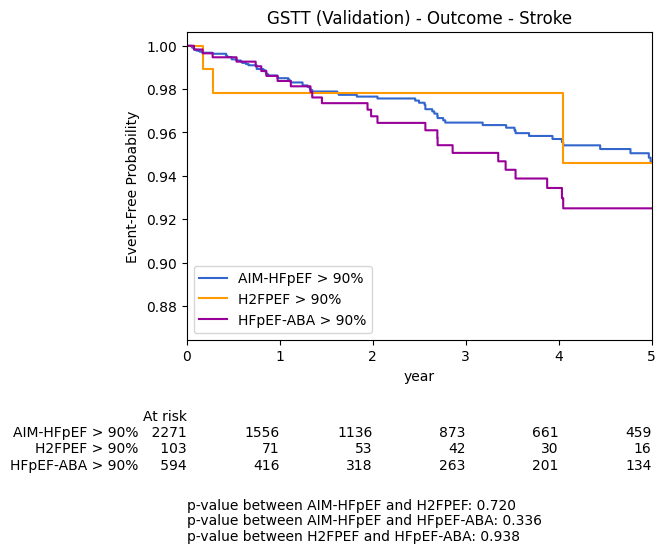 |
