# Supplementary material for: Artificial intelligence methods to detect heart failure with preserved ejection fraction within electronic health records: an equitable disease detection model
Source: Eur Heart J Digit Health. 2025 Sep 16;7(1):ztaf107. doi: 10.1093/ehjdh/ztaf107 (PMC12821069; doi:10.1093/ehjdh/ztaf107)
Supplement: ztaf107_Supplementary_Data [file ztaf107_supplementary_data.zip › Supplementary_Table_1.docx]

**Supplementary Table 1. SNOMED-CT terms used in the study for the inclusion and exclusion criteria.**

All child terms are included by default. Inclusion criteria include HF, HFpEF and Dyspnoea. Exclusion criteria include severe valvular heart disease, hypertrophic cardiomyopathy, restrictive cardiomyopathy, constrictive pericarditis, and cardiac amyloidosis

| **Criteria** | **SNOMED-CT Term** | **ID** |
| --- | --- | --- |
| Inclusion | Heart failure (disorder) | 84114007 |
|  | Heart failure with normal ejection fraction (disorder) | 446221000 |
|  | Dyspnoea (finding) | 267036007 |
| Exclusion | Aortic valve stenosis (disorder)^*^ | 60573004 |
|  | Aortic valve regurgitation (disorder) ^*^ | 60234000 |
|  | Mitral valve stenosis (disorder) ^*^ | 79619009 |
|  | Mitral valve regurgitation (disorder) ^*^ | 48724000 |
|  | Pulmonic valve stenosis (disorder) ^*^ | 56786000 |
|  | Pulmonic valve regurgitation (disorder) ^*^ | 91434003 |
|  | Hypertrophic cardiomyopathy (disorder) | 233873004 |
|  | Senile cardiac amyloidosis (disorder) | 16573007 |
|  | Restrictive cardiomyopathy (disorder) | 415295002 |
|  | Constrictive pericarditis (disorder) | 85598007 |

* - the **severe** valvular heart disease patients are identified with the ‘severe’ keyword before the mentions of the SNOMED-CT terms.
