# Supplementary material for: Artificial intelligence methods to detect heart failure with preserved ejection fraction within electronic health records: an equitable disease detection model
Source: Eur Heart J Digit Health. 2025 Sep 16;7(1):ztaf107. doi: 10.1093/ehjdh/ztaf107 (PMC12821069; doi:10.1093/ehjdh/ztaf107)
Supplement: ztaf107_Supplementary_Data [file ztaf107_supplementary_data.zip › Supplementary_Table_2.docx]

**Supplementary Table 2. Performances of all machine learning models**

F – Full Model (30 features)

S – Simplified Model (10 features)

LR – Logistic Regression

RF – Random Forest

SVM – Support Vector Machine

XGB – XGBoost

H2FPEF-Point – Point-based H2FPEF score

KCH Testing Cohort (n=635)

| **Model** | **Accuracy** | **Precision** | **Recall** | **F1-Score** | **AUC [95% CI]** |
| --- | --- | --- | --- | --- | --- |
| F-LR | 0.7760 | 0.8094 | 0.8896 | 0.8476 | 0.8204 [0.7868-0.8540] |
| F-RF | 0.7382 | 0.7473 | 0.9459 | 0.8350 | 0.8472 [0.8161-0.8784] |
| F-SVM | 0.7918 | 0.8250 | 0.8919 | 0.8571 | 0.8386 [0.8063-0.8709] |
| F-XGB | **0.8186** | **0.8600** | 0.8851 | **0.8724** | **0.8910 [0.8665-0.9154]** |
| S-LR | 0.7634 | 0.7988 | 0.8851 | 0.8397 | 0.7962 [0.7603-0.8321] |
| S-RF | 0.7524 | 0.7540 | **0.9595** | 0.8444 | 0.8202 [0.7851-0.8553] |
| S-SVM | 0.7792 | 0.8115 | 0.8919 | 0.8498 | 0.8181 [0.7835-0.8527] |
| S-XGB | 0.7981 | 0.8465 | 0.8694 | 0.8578 | 0.8801 [0.8539-0.9063] |

KCH Testing Cohort – Comparison with H2FPEF (n=182)

| **Model** | **Accuracy** | **Precision** | **Recall** | **F1-Score** | **AUC [95% CI]** | ***P*-value** |
| --- | --- | --- | --- | --- | --- | --- |
| F-LR | 0.7935 | 0.8649 | 0.8767 | 0.8707 | 0.8398 [0.7764-0.9031] | 0.0753 |
| F-RF | 0.8043 | 0.8354 | 0.9384 | 0.8839 | 0.8600 [0.8060-0.9141] | 0.0318 |
| F-SVM | 0.7989 | 0.8658 | 0.8836 | 0.8746 | 0.8506 [0.7844-0.9168] | 0.0447 |
| F-XGB | 0.8261 | 0.8800 | 0.9041 | 0.8919 | **0.8756 [0.8245-0.9267]** | 0.0062 |
| S-LR | 0.8043 | 0.8667 | 0.8904 | 0.8784 | 0.8145 [0.7443-0.8848] | 0.2289 |
| S-RF | 0.8098 | 0.8208 | **0.9726** | 0.8903 | 0.8278 [0.7579-0.8976] | 0.2122 |
| S-SVM | 0.8152 | 0.8636 | 0.9110 | 0.8867 | 0.8169 [0.7420-0.8917] | 0.2552 |
| S-XGB | 0.8261 | 0.8701 | 0.9178 | **0.8933** | 0.8610 [0.8040-0.9181] | 0.0136 |
| H2FPEF | 0.6848 | **0.9231** | 0.6575 | 0.7680 | 0.7873 [0.7075-0.8672] | - |
| H2FPEF-Point | **0.8152** | 0.8544 | 0.9247 | 0.8882 | 0.7222 [0.6259-0.8184] | 0.1663 |

KCH Testing Cohort – Comparison with HFpEF-ABA (n=483)

| **Model** | **Accuracy** | **Precision** | **Recall** | **F1-Score** | **AUC [95% CI]** | ***P*-value** |
| --- | --- | --- | --- | --- | --- | --- |
| F-LR | 0.7734 | 0.8150 | 0.8837 | 0.8480 | 0.8206 [0.7821-0.8591] | <0.0001 |
| F-RF | 0.7443 | 0.7552 | 0.9506 | 0.8417 | 0.8454 [0.8096-0.8811] | <0.0001 |
| F-SVM | 0.7879 | 0.8288 | 0.8866 | 0.8567 | 0.8353 [0.7973-0.8732] | <0.0001 |
| F-XGB | **0.8170** | **0.8657** | 0.8808 | **0.8732** | **0.8873 [0.8587-0.9159]** | <0.0001 |
| S-LR | 0.7588 | 0.8048 | 0.8750 | 0.8384 | 0.7883 [0.7460-0.8307] | 0.0063 |
| S-RF | 0.7464 | 0.7523 | **0.9622** | 0.8444 | 0.8156 [0.7751-0.8560] | 0.0005 |
| S-SVM | 0.7755 | 0.8155 | 0.8866 | 0.8496 | 0.8122 [0.7717-0.8527] | 0.0004 |
| S-XGB | 0.7963 | 0.8534 | 0.8634 | 0.8584 | 0.8729 [0.8420-0.9038] | <0.0001 |
| HFpEF-ABA | 0.7173 | 0.8230 | 0.7703 | 0.7958 | 0.7425 [0.6949-0.7901] | - |

KCH Testing Cohort – Non-White (n=202)

| **Model** | **Accuracy** | **Precision** | **Recall** | **F1-Score** | **AUC [95% CI]** |
| --- | --- | --- | --- | --- | --- |
| F-LR | 0.7824 | 0.8108 | 0.8955 | 0.8511 | 0.8327 [0.7715-0.8938] |
| F-RF | 0.7668 | 0.7633 | 0.9627 | 0.8515 | 0.8576 [0.8044-0.9108] |
| F-SVM | 0.7927 | 0.8264 | 0.8881 | 0.8561 | 0.8443 [0.7852-0.9034] |
| F-XGB | **0.8135** | **0.8451** | 0.8955 | **0.8696** | **0.8848 [0.8390-0.9305]** |
| S-LR | 0.7617 | 0.7973 | 0.8806 | 0.8369 | 0.8027 [0.7385-0.8668] |
| S-RF | 0.7824 | 0.7674 | **0.9851** | 0.8627 | 0.8134 [0.7461-0.8806] |
| S-SVM | 0.7565 | 0.7881 | 0.8881 | 0.8351 | 0.8058 [0.7420-0.8697] |
| S-XGB | 0.7927 | 0.8357 | 0.8731 | 0.8540 | 0.8725 [0.8228-0.9222] |

KCH Testing Cohort – Non-White – Comparison with H2FPEF (n=74)

| **Model** | **Accuracy** | **Precision** | **Recall** | **F1-Score** | **AUC [95% CI]** | ***P*-value** |
| --- | --- | --- | --- | --- | --- | --- |
| F-LR | 0.7973 | 0.8909 | 0.8448 | 0.8673 | 0.8513 [0.7472-0.9554] | 0.1586 |
| F-RF | 0.8243 | 0.8571 | 0.9310 | 0.8926 | 0.8594 [0.7721-0.9467] | 0.1175 |
| F-SVM | 0.8108 | 0.9074 | 0.8448 | 0.8750 | 0.8524 [0.7477-0.9571] | 0.1786 |
| F-XGB | 0.8108 | 0.8793 | 0.8793 | 0.8793 | **0.8739 [0.7899-0.9580]** | 0.0614 |
| S-LR | 0.7838 | 0.8750 | 0.8448 | 0.8596 | 0.8297 [0.7241-0.9354] | 0.3459 |
| S-RF | 0.8378 | 0.8382 | **0.9828** | **0.9048** | 0.8518 [0.7527-0.9509] | 0.2282 |
| S-SVM | 0.7838 | 0.8621 | 0.8621 | 0.8621 | 0.8308 [0.7281-0.9336] | 0.3952 |
| S-XGB | **0.8378** | 0.8966 | 0.8966 | 0.8966 | 0.8675 [0.7739-0.9610] | 0.1409 |
| H2FPEF | 0.6622 | **0.9459** | 0.6034 | 0.7368 | 0.7899 [0.6774-0.9023] | - |
| H2FPEF-Point | 0.7703 | 0.8154 | 0.9138 | 0.8618 | 0.6546 [0.4886-0.8207] | 0.1400 |

KCH Testing Cohort – Non-White – Comparison with HFpEF-ABA (n=159)

| **Model** | **Accuracy** | **Precision** | **Recall** | **F1-Score** | **AUC [95% CI]** | ***P*-value** |
| --- | --- | --- | --- | --- | --- | --- |
| F-LR | 0.7707 | 0.8197 | 0.8772 | 0.8475 | 0.8027 [0.7268-0.8787] | 0.2133 |
| F-RF | 0.7707 | 0.7786 | 0.9561 | 0.8583 | 0.8355 [0.7721-0.8988] | 0.0420 |
| F-SVM | 0.7834 | 0.8390 | 0.8684 | 0.8534 | 0.8154 [0.7408-0.8900] | 0.1153 |
| F-XGB | **0.8089** | 0.8559 | 0.8860 | **0.8707** | **0.8694 [0.8142-0.9247]** | 0.0020 |
| S-LR | 0.7580 | 0.8167 | 0.8596 | 0.8376 | 0.7746 [0.6963-0.8529] | 0.5567 |
| S-RF | 0.7834 | 0.7778 | **0.9825** | 0.8682 | 0.7841 [0.7025-0.8656] | 0.4250 |
| S-SVM | 0.7516 | 0.7953 | 0.8860 | 0.8382 | 0.7862 [0.7118-0.8607] | 0.3673 |
| S-XGB | 0.7898 | 0.8462 | 0.8684 | 0.8571 | 0.8570 [0.7978-0.9162] | 0.0059 |
| HFpEF-ABA | 0.7197 | **0.8571** | 0.7368 | 0.7925 | 0.7542 [0.6725-0.8359] | - |

KCH Testing Cohort – Low IMD (n=118)

| **Model** | **Accuracy** | **Precision** | **Recall** | **F1-Score** | **AUC [95% CI]** |
| --- | --- | --- | --- | --- | --- |
| F-LR | 0.7812 | 0.8447 | 0.8788 | 0.8614 | 0.8213 [0.7416-0.9010] |
| F-RF | 0.8125 | 0.8319 | 0.9495 | 0.8868 | 0.8467 [0.7745-0.9190] |
| F-SVM | 0.8047 | 0.8700 | 0.8788 | 0.8744 | 0.8746 [0.8151-0.9341] |
| F-XGB | **0.8438** | **0.8990** | 0.8990 | **0.8990** | **0.9011 [0.8460-0.9562]** |
| S-LR | 0.8047 | 0.8627 | 0.8889 | 0.8756 | 0.8036 [0.7223-0.8848] |
| S-RF | 0.8203 | 0.8276 | **0.9697** | 0.8930 | 0.8178 [0.7292-0.9065] |
| S-SVM | 0.7969 | 0.8544 | 0.8889 | 0.8713 | 0.8408 [0.7680-0.9136] |
| S-XGB | 0.8281 | 0.8969 | 0.8788 | 0.8878 | 0.8945 [0.8373-0.9516] |

KCH Testing Cohort – Low IMD – Comparison with H2FPEF (n=44)

| **Model** | **Accuracy** | **Precision** | **Recall** | **F1-Score** | **AUC [95% CI]** | ***P*-value** |
| --- | --- | --- | --- | --- | --- | --- |
| F-LR | 0.7727 | 0.9143 | 0.8205 | 0.8649 | 0.8359 [0.7065-0.9653] | 0.3105 |
| F-RF | 0.8409 | 0.8810 | 0.9487 | 0.9136 | 0.8077 [0.6738-0.9416] | 0.5139 |
| F-SVM | 0.7727 | 0.9143 | 0.8205 | 0.8649 | 0.8513 [0.7404-0.9622] | 0.1805 |
| F-XGB | 0.8409 | 0.9000 | 0.9231 | 0.9114 | **0.8615 [0.7282-0.9948]** | 0.2082 |
| S-LR | 0.8182 | 0.9429 | 0.8462 | 0.8919 | 0.8359 [0.6985-0.9732] | 0.2904 |
| S-RF | 0.8636 | 0.8837 | **0.9744** | 0.9268 | 0.8436 [0.7111-0.9761] | 0.2632 |
| S-SVM | 0.8182 | 0.9189 | 0.8718 | 0.8947 | 0.8821 [0.7763-0.9878] | 0.0523 |
| S-XGB | **0.9091** | 0.9487 | 0.9487 | **0.9487** | 0.8615 [0.6759-1.0000] | 0.3101 |
| H2FPEF | 0.6818 | **1.0000** | 0.6410 | 0.7812 | 0.7538 [0.6080-0.8996] | - |
| H2FPEF-Point | 0.7955 | 0.9167 | 0.8462 | 0.8800 | 0.6821 [0.3958-0.9683] | 0.5871 |

KCH Testing Cohort – Low IMD – Comparison with HFpEF-ABA (n=93)

| **Model** | **Accuracy** | **Precision** | **Recall** | **F1-Score** | **AUC [95% CI]** | ***P*-value** |
| --- | --- | --- | --- | --- | --- | --- |
| F-LR | 0.7660 | 0.8421 | 0.8649 | 0.8533 | 0.8034 [0.7023-0.9044] | 0.3102 |
| F-RF | 0.7979 | 0.8161 | 0.9595 | 0.8820 | 0.8007 [0.7034-0.8980] | 0.2763 |
| F-SVM | 0.7872 | 0.8553 | 0.8784 | 0.8667 | 0.8500 [0.7728-0.9272] | 0.0319 |
| F-XGB | **0.8404** | **0.8831** | 0.9189 | **0.9007** | **0.8892 [0.8185-0.9599]** | 0.0098 |
| S-LR | 0.7979 | 0.8667 | 0.8784 | 0.8725 | 0.7682 [0.6630-0.8735] | 0.5727 |
| S-RF | 0.8191 | 0.8202 | **0.9865** | 0.8957 | 0.7784 [0.6601-0.8967] | 0.4318 |
| S-SVM | 0.7766 | 0.8442 | 0.8784 | 0.8609 | 0.8182 [0.7238-0.9127] | 0.1071 |
| S-XGB | 0.8298 | 0.8816 | 0.9054 | 0.8933 | 0.8818 [0.8075-0.9560] | 0.0107 |
| HFpEF-ABA | 0.7234 | 0.8636 | 0.7703 | 0.8143 | 0.7426 [0.6245-0.8607] | - |

GSTT Validation Cohort (n=5,383)

| **Model** | **Accuracy** | **Precision** | **Recall** | **F1-Score** | **AUC [95% CI]** |
| --- | --- | --- | --- | --- | --- |
| F-LR | 0.7251 | 0.7364 | 0.8692 | 0.7973 | 0.7989 [0.7870-0.8108] |
| F-RF | 0.7485 | 0.7340 | 0.9343 | 0.8221 | 0.8348 [0.8237-0.8459] |
| F-SVM | 0.7377 | 0.7481 | 0.8719 | 0.8053 | 0.8086 [0.7970-0.8203] |
| F-XGB | **0.7986** | **0.8112** | 0.8815 | **0.8449** | **0.8934 [0.8852-0.9016]** |
| S-LR | 0.7265 | 0.7237 | 0.9065 | 0.8049 | 0.8189 [0.8076-0.8302] |
| S-RF | 0.7236 | 0.7071 | **0.9486** | 0.8103 | 0.8327 [0.8217-0.8438] |
| S-SVM | 0.7271 | 0.7221 | 0.9125 | 0.8062 | 0.8075 [0.7954-0.8196] |
| S-XGB | 0.7866 | 0.7924 | 0.8901 | 0.8384 | 0.8900 [0.8817-0.8983] |

GSTT Validation Cohort – Comparison with H2FPEF (n=914)

| **Model** | **Accuracy** | **Precision** | **Recall** | **F1-Score** | **AUC [95% CI]** | ***P*-value** |
| --- | --- | --- | --- | --- | --- | --- |
| F-LR | 0.6685 | 0.5817 | 0.8223 | 0.6814 | 0.7849 [0.7550-0.8149] | 0.6847 |
| F-RF | 0.6411 | 0.5493 | 0.9340 | 0.6917 | 0.8242 [0.7972-0.8513] | 0.0001 |
| F-SVM | 0.6783 | 0.5871 | 0.8553 | 0.6963 | 0.8016 [0.7726-0.8306] | 0.0623 |
| F-XGB | 0.7123 | 0.6105 | 0.9188 | **0.7335** | **0.8821 [0.8596-0.9047]** | <0.0001 |
| S-LR | 0.6586 | 0.5681 | 0.8680 | 0.6867 | 0.7892 [0.7595-0.8188] | 0.2458 |
| S-RF | 0.5755 | 0.5041 | 0.9365 | 0.6554 | 0.8065 [0.7777-0.8352] | 0.0138 |
| S-SVM | 0.6061 | 0.5257 | 0.8832 | 0.6591 | 0.7826 [0.7519-0.8133] | 0.8577 |
| S-XGB | 0.6575 | 0.5615 | **0.9391** | 0.7028 | 0.8808 [0.8578-0.9038] | <0.0001 |
| H2FPEF | **0.7144** | **0.6692** | 0.6675 | 0.6684 | 0.7805 [0.7505-0.8105] | - |
| H2FPEF-Point | 0.6772 | 0.5980 | 0.7665 | 0.6719 | 0.7550 [0.7242-0.7858] | 0.0072 |

GSTT Validation Cohort – Comparison with HFpEF-ABA (n=3,497)

| **Model** | **Accuracy** | **Precision** | **Recall** | **F1-Score** | **AUC [95% CI]** | ***P*-value** |
| --- | --- | --- | --- | --- | --- | --- |
| F-LR | 0.6391 | 0.5440 | 0.8496 | 0.6633 | 0.7800 [0.7644-0.7956] | 0.0119 |
| F-RF | 0.6446 | 0.5442 | 0.9255 | 0.6854 | 0.8236 [0.8098-0.8375] | <0.0001 |
| F-SVM | 0.6597 | 0.5610 | 0.8585 | 0.6786 | 0.7949 [0.7798-0.8101] | <0.0001 |
| F-XGB | **0.7518** | **0.6511** | 0.8763 | **0.7471** | **0.8898 [0.8784-0.9012]** | <0.0001 |
| S-LR | 0.6237 | 0.5298 | 0.8927 | 0.6650 | 0.8009 [0.7860-0.8158] | <0.0001 |
| S-RF | 0.5979 | 0.5106 | **0.9385** | 0.6614 | 0.8203 [0.8060-0.8345] | <0.0001 |
| S-SVM | 0.6200 | 0.5270 | 0.8954 | 0.6635 | 0.7987 [0.7836-0.8138] | <0.0001 |
| S-XGB | 0.7303 | 0.6249 | 0.8893 | 0.7340 | 0.8880 [0.8765-0.8996] | <0.0001 |
| HFpEF-ABA | 0.6623 | 0.5687 | 0.7977 | 0.6640 | 0.7624 [0.7462-0.7787] | - |

GSTT Validation Cohort – Non-White (n=1,186)

| **Model** | **Accuracy** | **Precision** | **Recall** | **F1-Score** | **AUC [95% CI]** |
| --- | --- | --- | --- | --- | --- |
| F-LR | 0.7125 | 0.7112 | 0.8610 | 0.7790 | 0.7956 [0.7703-0.8210] |
| F-RF | 0.7302 | 0.7019 | **0.9413** | 0.8042 | 0.8301 [0.8066-0.8536] |
| F-SVM | 0.7218 | 0.7201 | 0.8625 | 0.7849 | 0.8013 [0.7764-0.8261] |
| F-XGB | **0.7850** | **0.7814** | 0.8811 | **0.8283** | **0.8893 [0.8717-0.9070]** |
| S-LR | 0.7066 | 0.6957 | 0.8911 | 0.7814 | 0.8025 [0.7779-0.8271] |
| S-RF | 0.6897 | 0.6684 | 0.9384 | 0.7807 | 0.8200 [0.7962-0.8438] |
| S-SVM | 0.6965 | 0.6861 | 0.8926 | 0.7758 | 0.7949 [0.7691-0.8207] |
| S-XGB | 0.7639 | 0.7568 | 0.8825 | 0.8148 | 0.8782 [0.8595-0.8969] |

GSTT Validation Cohort – Non-White – Comparison with H2FPEF (n=228)

| **Model** | **Accuracy** | **Precision** | **Recall** | **F1-Score** | **AUC [95% CI]** | ***P*-value** |
| --- | --- | --- | --- | --- | --- | --- |
| F-LR | 0.7018 | 0.6439 | 0.8019 | 0.7143 | 0.7877 [0.7284-0.8469] | 0.4246 |
| F-RF | 0.6798 | 0.5976 | 0.9528 | 0.7345 | 0.8304 [0.7786-0.8823] | 0.0119 |
| F-SVM | 0.7149 | 0.6414 | 0.8774 | 0.7410 | 0.8017 [0.7447-0.8588] | 0.1444 |
| F-XGB | **0.7412** | 0.6556 | 0.9340 | **0.7704** | **0.9041 [0.8666-0.9416]** | <0.0001 |
| S-LR | 0.6886 | 0.6190 | 0.8585 | 0.7194 | 0.7868 [0.7285-0.8451] | 0.2467 |
| S-RF | 0.5877 | 0.5319 | 0.9434 | 0.6803 | 0.7930 [0.7341-0.8518] | 0.2681 |
| S-SVM | 0.6360 | 0.5714 | 0.8679 | 0.6891 | 0.7709 [0.7086-0.8332] | 0.9073 |
| S-XGB | 0.6842 | 0.5988 | **0.9717** | 0.7410 | 0.8958 [0.8559-0.9357] | <0.0001 |
| H2FPEF | 0.6974 | **0.7033** | 0.6038 | 0.6497 | 0.7681 [0.7071-0.8291] | - |
| H2FPEF-Point | 0.6711 | 0.6260 | 0.7264 | 0.6725 | 0.7394 [0.6760-0.8028] | 0.1688 |

GSTT Validation Cohort – Non-White – Comparison with HFpEF-ABA (n=827)

| **Model** | **Accuracy** | **Precision** | **Recall** | **F1-Score** | **AUC [95% CI]** | ***P*-value** |
| --- | --- | --- | --- | --- | --- | --- |
| F-LR | 0.6312 | 0.5326 | 0.8201 | 0.6458 | 0.7678 [0.7347-0.8010] | 0.0003 |
| F-RF | 0.6409 | 0.5350 | **0.9469** | 0.6837 | 0.8219 [0.7937-0.8500] | <0.0001 |
| F-SVM | 0.6590 | 0.5543 | 0.8584 | 0.6736 | 0.7898 [0.7581-0.8216] | <0.0001 |
| F-XGB | **0.7497** | **0.6387** | 0.8968 | **0.7460** | **0.8990 [0.8768-0.9212]** | <0.0001 |
| S-LR | 0.6179 | 0.5203 | 0.8702 | 0.6512 | 0.7767 [0.7444-0.8089] | <0.0001 |
| S-RF | 0.5792 | 0.4930 | 0.9322 | 0.6449 | 0.8059 [0.7754-0.8364] | <0.0001 |
| S-SVM | 0.6010 | 0.5078 | 0.8673 | 0.6405 | 0.7769 [0.7444-0.8094] | <0.0001 |
| S-XGB | 0.7158 | 0.6040 | 0.8909 | 0.7199 | 0.8873 [0.8630-0.9116] | <0.0001 |
| HFpEF-ABA | 0.6518 | 0.5609 | 0.6932 | 0.6201 | 0.7101 [0.6734-0.7468] | - |

GSTT Validation Cohort – Low IMD (n=1,091)

| **Model** | **Accuracy** | **Precision** | **Recall** | **F1-Score** | **AUC [95% CI]** |
| --- | --- | --- | --- | --- | --- |
| F-LR | 0.7324 | 0.7528 | 0.8670 | 0.8059 | 0.8074 [0.7816-0.8332] |
| F-RF | 0.7736 | 0.7580 | **0.9499** | 0.8432 | 0.8513 [0.8276-0.8751] |
| F-SVM | 0.7544 | 0.7731 | 0.8727 | 0.8199 | 0.8202 [0.7949-0.8454] |
| F-XGB | **0.8130** | **0.8322** | 0.8870 | **0.8587** | **0.8969 [0.8789-0.9148]** |
| S-LR | 0.7406 | 0.7441 | 0.9070 | 0.8175 | 0.8358 [0.8117-0.8599] |
| S-RF | 0.7360 | 0.7251 | 0.9471 | 0.8213 | 0.8469 [0.8232-0.8706] |
| S-SVM | 0.7415 | 0.7467 | 0.9027 | 0.8174 | 0.8114 [0.7838-0.8390] |
| S-XGB | 0.8084 | 0.8224 | 0.8941 | 0.8568 | 0.8964 [0.8786-0.9142] |

GSTT Validation Cohort – Low IMD – Comparison with H2FPEF (n=184)

| **Model** | **Accuracy** | **Precision** | **Recall** | **F1-Score** | **AUC [95% CI]** | ***P*-value** |
| --- | --- | --- | --- | --- | --- | --- |
| F-LR | 0.6902 | 0.6667 | 0.7609 | 0.7107 | 0.7811 [0.7133-0.8488] | 0.9449 |
| F-RF | 0.6957 | 0.6364 | 0.9130 | 0.7500 | 0.8355 [0.7751-0.8959] | 0.0365 |
| F-SVM | 0.7609 | 0.7264 | 0.8370 | 0.7778 | 0.8358 [0.7762-0.8954] | 0.0323 |
| F-XGB | **0.7935** | **0.7411** | 0.9022 | **0.8137** | 0.8982 [0.8505-0.9459] | 0.0001 |
| S-LR | 0.7011 | 0.6555 | 0.8478 | 0.7393 | 0.7948 [0.7294-0.8601] | 0.3598 |
| S-RF | 0.6033 | 0.5646 | 0.9022 | 0.6946 | 0.8116 [0.7477-0.8754] | 0.2355 |
| S-SVM | 0.6467 | 0.6047 | 0.8478 | 0.7059 | 0.7828 [0.7135-0.8522] | 0.9028 |
| S-XGB | 0.7446 | 0.6772 | **0.9348** | 0.7854 | **0.8990 [0.8519-0.9460]** | <0.0001 |
| H2FPEF | 0.7065 | 0.7317 | 0.6522 | 0.6897 | 0.7793 [0.7127-0.8459] | - |
| H2FPEF-Point | 0.7065 | 0.6759 | 0.7935 | 0.7300 | 0.7517 [0.6819-0.8214] | 0.1754 |

GSTT Validation Cohort – Low IMD – Comparison with HFpEF-ABA (n=681)

| **Model** | **Accuracy** | **Precision** | **Recall** | **F1-Score** | **AUC [95% CI]** | ***P*-value** |
| --- | --- | --- | --- | --- | --- | --- |
| F-LR | 0.6388 | 0.5488 | 0.8374 | 0.6630 | 0.7761 [0.7399-0.8123] | 0.9205 |
| F-RF | 0.6608 | 0.5602 | **0.9343** | 0.7004 | 0.8365 [0.8064-0.8665] | <0.0001 |
| F-SVM | 0.6799 | 0.5828 | 0.8651 | 0.6964 | 0.8058 [0.7721-0.8395] | 0.0459 |
| F-XGB | **0.7709** | **0.6736** | 0.8927 | **0.7679** | **0.8993 [0.8749-0.9237]** | <0.0001 |
| S-LR | 0.6314 | 0.5401 | 0.8858 | 0.6710 | 0.8101 [0.7765-0.8438] | 0.0015 |
| S-RF | 0.6006 | 0.5164 | 0.9273 | 0.6634 | 0.8256 [0.7939-0.8572] | 0.0005 |
| S-SVM | 0.6373 | 0.5447 | 0.8858 | 0.6746 | 0.8013 [0.7671-0.8355] | 0.0490 |
| S-XGB | 0.7577 | 0.6574 | 0.8962 | 0.7584 | 0.8981 [0.8735-0.9227] | <0.0001 |
| HFpEF-ABA | 0.6711 | 0.5799 | 0.8166 | 0.6782 | 0.7745 | - |
