# Supplementary material for: Artificial intelligence methods to detect heart failure with preserved ejection fraction within electronic health records: an equitable disease detection model
Source: Eur Heart J Digit Health. 2025 Sep 16;7(1):ztaf107. doi: 10.1093/ehjdh/ztaf107 (PMC12821069; doi:10.1093/ehjdh/ztaf107)
Supplement: ztaf107_Supplementary_Data [file ztaf107_supplementary_data.zip › Supplementary_Table_3.docx]

**Supplementary Table 3. SHAP values of the top features.**

| **Feature** | **Mean \|SHAP value\|** |
| --- | --- |
| NTproBNP | 0.8559 |
| Age | 0.5352 |
| PASP | 0.4942 |
| LA volume | 0.2055 |
| BMI | 0.1512 |
| Atrial fibrillation | 0.1216 |
| Sex | 0.1137 |
| LV mass | 0.1105 |
| Chest pain | 0.1080 |
| Type 2 diabetes mellitus | 0.1009 |
