# Supplementary figures and images for: Artificial intelligence methods to detect heart failure with preserved ejection fraction within electronic health records: an equitable disease detection model
Source: Eur Heart J Digit Health. 2025 Sep 16;7(1):ztaf107. doi: 10.1093/ehjdh/ztaf107 (PMC12821069; doi:10.1093/ehjdh/ztaf107)

**Supplementary Figure 6.** Prevalence of H2FPEF Features in Confirmed and ESC Criteria HFpEF Cases


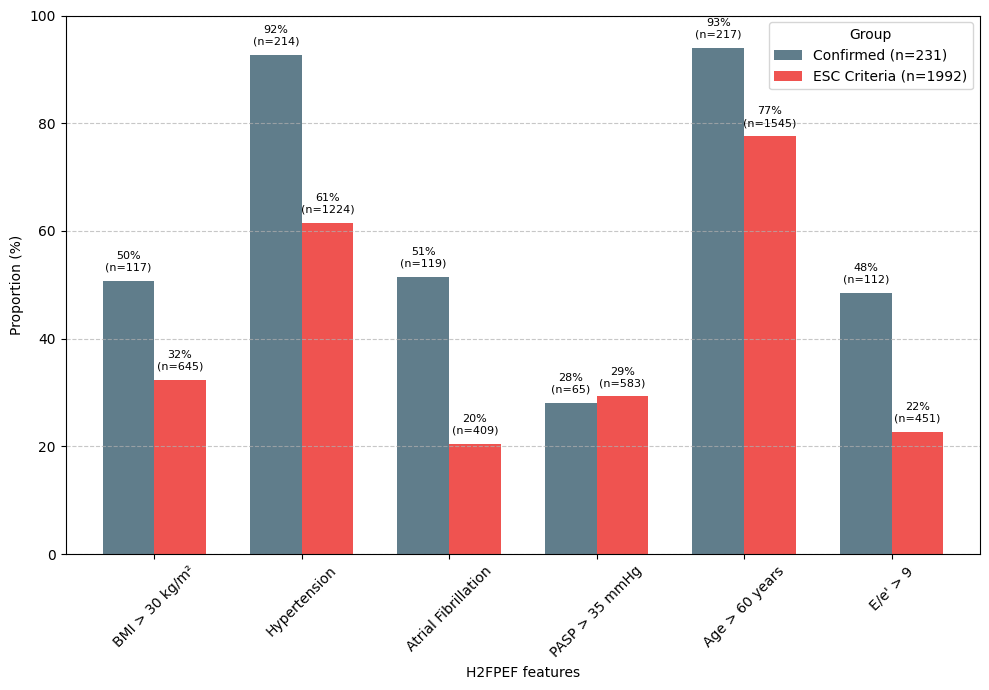

Supplement: ztaf107_Supplementary_Data [file ztaf107_supplementary_data.zip › Supplementary_Figure_6.docx]
